# Effect of neonatal and adult sepsis on inflammation-related diseases in multiple physiological systems: a Mendelian randomization study

**DOI:** 10.3389/fendo.2023.1215751

**Published:** 2023-07-20

**Authors:** Suping Li, Qian Wang, Xin Tan, Linghua Wang, Jin Gong, Juan Zhang, Weilin Wang, Jiangling Liu

**Affiliations:** ^1^ Department of Neonatal Intensive Care Unit, Hunan Provincial Maternal and Child Health Care Hospital, Changsha, Hunan, China; ^2^ Department of Pediatrics, The First Hospital of Changsha, Changsha, Hunan, China

**Keywords:** adult, body mass index, neonate, obesity, sepsis

## Abstract

**Background:**

Long-term impact of sepsis on whole body systems is not well investigated. The aim of the study was to explore the potential association of neonatal/adult sepsis with several inflammation-related diseases in multiple physiological systems.

**Methods:**

Instrumental variables for neonatal and adult sepsis were collected from the public genome-wide association studies, which must satisfy the correlation, exclusivity and independence assumptions. Mendelian randomization methods (including random-effect inverse-variance weighted, MR-PRESSO, weighted median and MR-Egger) were used to determine the genetic association of neonatal/adult sepsis with asthma, allergy, rheumatoid arthritis, body mass index/obesity, type 1/type 2 diabetes and intelligence/dementia. Sensitivity analyses were conducted to assess heterogeneity and horizontal pleiotropy. The study was performed by TwoSampleMR in R software.

**Results:**

The inverse-variance weighted method reported that neonatal sepsis was related to the decreased level of body mass index (OR = 0.988, 95%CI = 0.980 ~ 0.997, P = 0.007), and adult sepsis was related to the decreased risk of obesity (OR = 0.785, 95%CI = 0.655 ~ 0.940, P = 0.009). These results were supported by the other Mendelian randomization methods. In addition, the study did not find any association of neonatal/adult sepsis with the other inflammation-related diseases. No heterogeneity and horizontal pleiotropy were found using sensitivity analyses.

**Conclusion:**

Sepsis had the potential to reduce the risk of obesity or body mass index level at a genetic level, both in neonates and in adults.

## 1 Introduction

In 2017, about 48.9 million people suffered from sepsis globally, and resulted in 11 million of them deaths (1). In the same year, nearly 5 million people had sepsis in China, and about 1 million died of this disease (1, 2). People of all ages are at risk of developing sepsis. Specially, neonates are easily affected by it due to their immature immune function, while adults are also susceptible to the disease if they are immunocompromised (3, 4).

Sepsis is a systemic inflammatory process. Briefly, when a large number of pathogenic bacteria enter the circulation and multiply in the blood, a systemic inflammatory response may be triggered, and causes widespread damage to numerous physiological systems in the body (5, 6). Meanwhile, inflammatory response is implicated in many common diseases in several physiological systems of human beings. For example, asthma, allergies, rheumatoid arthritis and type 1 diabetes themselves are regarded to be inflammatory or immune disorders (7–10). Pathogeneses of obesity and type 2 diabetes are closely linked to low-degree metabolic inflammation throughout the body (11, 12). Inflammatory response may also affect the development of intelligence in minors and contribute to the occurrence of cognitive impairment in adults (13, 14). However, it remains unclear whether sepsis has an effect on these inflammation-related diseases in different age groups.

A number of observational studies have explored these issues mentioned above. For example, Cetinkaya et al. suggested that exposure to severe infections such as sepsis in the neonatal period may decrease sensitization to environmental allergens and prevalence of asthma in later childhood (15). Rivas et al. concluded that insulin sensitivity and blood glucose levels were significantly disturbed during sepsis, but the associated long-term effects remain to be explored (16). Jiang et al. reported that sepsis may affect neurodevelopment by altering the concentration of proteins in the cerebrospinal fluid, with long-term adverse consequences for neonates (17). Lei et al. in their meta-analysis found that sepsis survivals were associated with an increased risk of all-cause dementia, and appropriate management and prevention were essential to preserve the cognitive function in this population (18). These results provide some evidence for the effect of sepsis on inflammation-related diseases in multiple physiological systems. However, the limitations of observational studies prevent a firmed conclusion to be drawn, so the issues should be supported by higher level evidence-based medical research.

Therefore, this study adopted summary data from published genome-wide association studies (GWASs) to conduct a series of MR analyses for the genetic effect of neonatal and adult sepsis on several inflammation-related diseases (i.e. asthma, allergy, rheumatoid arthritis, obesity/body mass index, type 1/2 diabetes and intelligence/dementia) in multiple physiological systems. Thus, this study might broadly add to our understanding of the long-term effect of sepsis on the whole body in patients of different ages.

## 2 Methods

### 2.1 Summary data for neonatal sepsis and its outcomes

The summary data for neonatal sepsis was collected from the most updated GWAS of this disease which included 224 late-onset neonatal sepsis cases and 273 controls from six European countries (19). In this GWAS, late-onset neonatal sepsis was defined as sepsis presenting from 3 to 90 days of age, and the diagnosis was established by clinical criteria consensus guidelines.

The summary data for asthma, allergy, juvenile rheuma and type 1 diabetes were obtained from the FinnGen studies in IEU OpenGWAS project (https://gwas.mrcieu.ac.uk/) using 138,474 to 218,792 Europeans. Subjects with asthma or allergies were younger than 16 years old, while the mean age at first presentation was 21 and 14 years for juvenile rheuma and type 1 diabetes, respectively. These diagnoses were established using ICD-10 codes in hospitalization records. For asthma, the ICD-10 codes were J45 and J46. For allergy, the ICD-10 codes were J30.10, J30.19, J30.2, J30.3, J30.4, J45.0, L20 and L23.6. For juvenile rheuma, the ICD-10 code was M08.0. For type 1 diabetes, the ICD-10 code was E10.

The summary data for body mass index was collected from a GWAS meta-analysis of body mass index in 39,620 European children aged between 2 and 10 years (20). The summary data for intelligence was obtained from the first GWAS on childhood intelligence from 12,441 European individuals aged between 6 and 18 years in six discovery and three replication samples (21).

Detailed information about these summary data were presented in **Table 1**.

**Table 1:** Sources of summary data in the study.

Traits	Sources	Years	Population	Gender	Age	Samplesize	No. of SNPs
Neonatal sepsis	PMID: 35835848	2022	European	Both	Mean: 22 days	497	11
Asthma	FinnGen	2021	European	Both	< 16 years	138,474	—
Allergy	FinnGen	2021	European	Both	< 16 years	218,792	—
Juvenile rheuma	FinnGen	2021	European	Both	Mean: 21 years ^a^	147,573	—
Body mass index	PMID: 33045005	2020	European	Both	2 ~ 10 years	39,620	—
Type 1 diabetes	FinnGen	2021	European	Both	Mean: 14 years ^a^	185,115	—
Intelligence	PMID: 23358156	2014	European	Both	6 ~ 18 years	12,441	—
Adult sepsis	PMID: 32966752	2020	European	Both	Adult	462,918	10
Asthma	FinnGen	2021	European	Both	Mean: 45 years ^a^	156,078	—
Allergy	FinnGen	2021	European	Both	Mean: 32 years ^a^	217436	—
Rheumatoid arthritis	FinnGen	2021	European	Both	Mean: 52 years ^a^	153,457	—
Obesity	FinnGen	2021	European	Both	Mean: 49 years ^a^	218,735	—
Type 2 diabetes	FinnGen	2021	European	Both	Mean: 59 years ^a^	215,654	—
Dementia	FinnGen	2021	European	Both	Mean: 78 years ^a^	216,771	—

^a^ Mean age at first event.

### 2.2 Summary data for adult sepsis and its outcomes

The summary data for adult sepsis was obtained from a MR Investigation including 462,918 adult Europeans (i.e. 10,154 sepsis cases and 454,764 controls) from UK Biobank, and the diagnosis was established by clinical criteria consensus guidelines (22).

The summary data for all the adult outcomes (i.e. asthma, allergy, rheumatoid arthritis, obesity, type 2 diabetes and dementia) were obtained from the FinnGen studies including 153,457 to 218,735 Europeans. The mean ages at first presentation for these diseases were 32 to 78 years. The cases were collected according to ICD-10 codes in hospitalization records or death records. For asthma, the ICD-10 codes were J45 and J46. For allergy, the ICD-10 codes were J30.10 and J30.19. For rheumatoid arthritis, the ICD-10 codes were M05 and M06. For obesity, the ICD-10 code was E66. For type 2 diabetes, the ICD-10 code was E11. For dementia, the ICD-10 codes were F00-F09.

Detailed information about these summary data were presented in **Table 1**.

### 2.3 Instrumental variables

Suitable single nucleotide polymorphisms (SNPs) in the study were collected from the above exposure-associated GWASs, and were called as instrumental variables. These SNPs must satisfy three main assumptions, namely the correlation assumption, exclusivity assumption and independence assumption. In order to meet these standards, the following measures were used: (1) The genome-wide significance P value for each SNP must be less than 5×10^−6^, but in the only published GWAS for neonatal sepsis worldwide, there was no SNPs that can meet this requirement, so the P value was relaxed to 5×10^−5^. P values at similar levels had been adopted in the previous MR study and yielded valuable results (23). (2) The F statistic for each SNP must be larger than 10 to ensure that it was not a weak instrumental variable. (3) The SNPs with linkage disequilibrium (LD) were removed directly using a clumping distance of 10 MB and r^2^ value of 0.001. (4) The SNPs that were significantly related to the outcomes or confounding factors were manually excluded using PhenoScanner (http://www.phenoscanner.medschl.cam.ac.uk/).

According to these requirements, eleven and ten SNPs were extracted from the published or publicly available GWASs mentioned above for predicting neonatal and adult sepsis in this study and participating in the following MR analyses, respectively (**Table 1**). In addition, the details of these SNPs were shown in **Supplementary Table 1**.

### 2.4 MR analyses

The random-effect inverse-variance weighted (IVW), MR-PRESSO, weighted median and MR-Egger were adopted to perform the MR analyses (24, 25). In the absence of any horizontal pleiotropic interference, IVW was the optimal method because it had the greatest ability to detect causality. The MR-PRESSO results were almost as accurate as the IVW results when the number of SNPs affected by horizontal pleiotropy was less than 10% of the total number. The other two methods can be used in the presence of pleiotropy. The weighted median allowed about 50% of instrumental variables to be pleiotropic, while the MR-Egger tolerated all instrumental variables to be pleiotropic, but only if these pleiotropies did not affect the correlation of instrumental variables with exposures. All four methods reported odds ratios (OR), 95% confidence intervals (95%CI) and P values. When an IVW P value was less than 0.05 and the results of the other three methods were in the same direction as the result of IVW, the causal correlation was considered statistically significant. Scatter plots and forest plots were presented to visualize the results.

Sensitivity analyses were also conducted in the study. First, the Cochran’s Q test was used to assess the heterogeneity. Second, the MR-Egger intercept test and funnel plot was adopted to determine the horizontal pleiotropy. Third, the MR-PRESSO test and leave-one-out test were used to remove the outliers, which was still related to the horizontal pleiotropy.

All analyses were performed by TwoSampleMR package in R software (version 4.2.2).

## 3 Results

### 3.1 Effect of neonatal sepsis on the inflammation-related diseases

**Figure 1:**
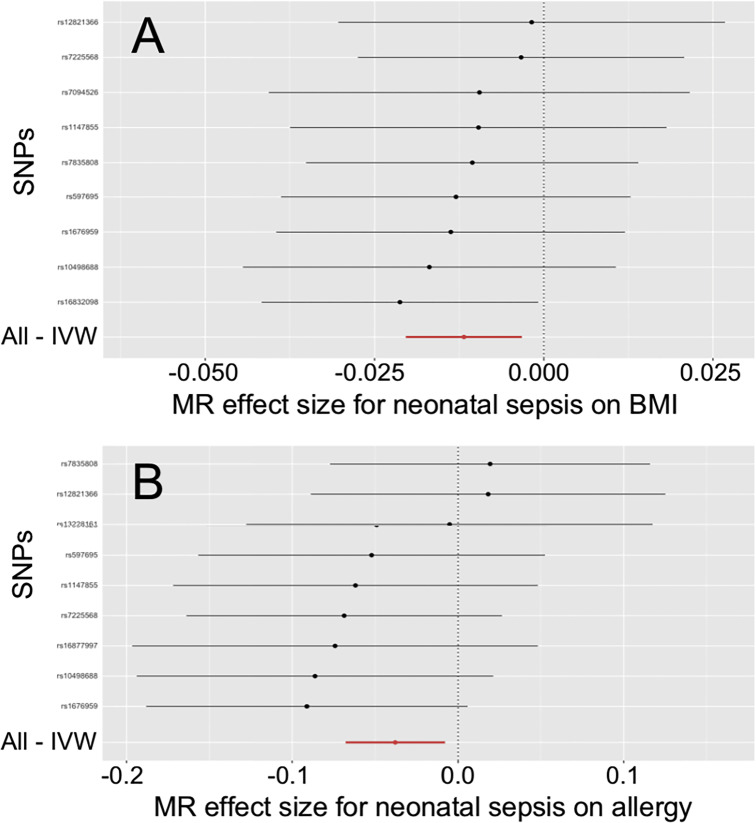
Effects of neonatal sepsis on body mass index and allergy. **(A)** Forest plot for the effect of neonatal sepsis on body mass index. **(B)** Forest plot for the effect of neonatal sepsis on allergy. SNP, Single nucleotide polymorphism; IVW, Inverse-variance weighted; MR, Mendelian randomization; BMI, Body mass index.

In **Table 2**, the IVW and MR-PRESSO reported that neonatal sepsis was significantly related to the decreased levels of body mass index (OR = 0.988, 95%CI = 0.980 ~ 0.997, P = 0.007; OR = 0.988, 95%CI = 0.984 ~ 0.992, P = 0.001). Although the weighted median and MR-Egger results were not significant, they were in the same direction as the IVW result. In **Figure 1A** and **Supplemental Figures 4A, B**, the scatter plot and forest plot visualized the above results. In **Table 2** and **Supplemental Figures 4C, D**, the Cochran’s Q test did not find significant heterogeneity (P = 0.982), the MR-Egger intercept and MR-PRESSO tests did not detect any horizontal pleiotropy and outliers (P = 0.585, P = 0.997), and the funnel plot and leave-one-out test also found no horizontal pleiotropy and outliers in these analyses.

**Table 2:** MR results for neonatal sepsis affecting inflammation-related diseases in multiple physiological systems.

Outcomes/ Methods	P_MR ^a^	ORs	95% CIs	P_CQ ^a^	P_IN ^a^	P_PR ^a^
Asthma						
IVW	0.683	0.993	0.962 ∼ 1.026	0.234	0.066	0.259
MR-PRESSO	0.692	0.993	0.962 ∼ 1.026			
Weighted median	0.711	0.991	0.942 ∼ 1.041			
MR Egger	0.088	1.119	0.997 ∼ 1.257			
Allergy						
**IVW**	**0.013**	**0.963**	**0.934 ∼ 0.992**	0.773	0.231	0.921
**MR-PRESSO**	**0.012**	**0.963**	**0.940 ∼ 0.986**			
Weighted median	0.879	0.994	0.921 ∼ 1.073			
MR Egger	0.541	1.040	0.921 ∼ 1.175			
Juvenile rheuma						
IVW	0.982	0.999	0.901 ∼ 1.107	0.069	0.314	0.092
MR-PRESSO	0.983	0.999	0.901 ∼ 1.107			
Weighted median	0.642	1.052	0.849 ∼ 1.303			
MR Egger	0.332	1.234	0.828 ∼ 1.838			
Body mass index						
**IVW**	**0.007**	**0.988**	**0.980 ∼ 0.997**	0.982	0.585	0.997
**MR-PRESSO**	**0.001**	**0.988**	**0.984 ∼ 0.992**			
Weighted median	0.236	0.992	0.978 ∼ 1.006			
MR Egger	0.282	0.978	0.941 ∼ 1.016			
Type 1 diabetes						
IVW	0.928	1.001	0.971 ∼ 1.033	0.709	0.805	0.724
MR-PRESSO	0.917	1.001	0.975 ∼ 1.028			
Weighted median	0.947	1.002	0.956 ∼ 1.049			
MR Egger	0.794	1.017	0.897 ∼ 1.154			
Intelligence						
IVW	0.651	1.004	0.986 ∼ 1.022	0.914	0.768	0.916
MR-PRESSO	0.446	1.004	0.994 ∼ 1.014			
Weighted median	0.920	0.998	0.968 ∼ 1.030			
MR Egger	0.715	1.020	0.923 ∼ 1.127			

^a^ “P_MR” indicated “P value for Mendelian randomization”; “P_CQ” indicated “P value for Cochran’s Q test”; “P_IN” indicated “MR-Egger intercept test”; “P_PR” indicated “MR-PRESSO for horizontal pleiotropy”. Bold font indicates that the results are statistically significant (P<0.05).

The IVW and MR-PRESSO also reported that neonatal sepsis was significantly related to the decreased risk of allergy (OR = 0.963, 95%CI = 0.934 ~ 0.992, P = 0.013; OR = 0.963, 95%CI = 0.940 ~ 0.986, P = 0.012). However, the MR-Egger result was not consistent with the IVW result in terms of direction. In addition, the study did not find any association of neonatal sepsis with the other inflammation-related diseases. These results were showed in **Table 2**. The scatter plots, forest plots, funnel plots and leave-one-out tests for all these results in the section were presented in **Figure 1** and **Supplementary Figures 1-6**.

### 3.2 Effect of adult sepsis on the inflammation-related diseases

**Figure 2:**
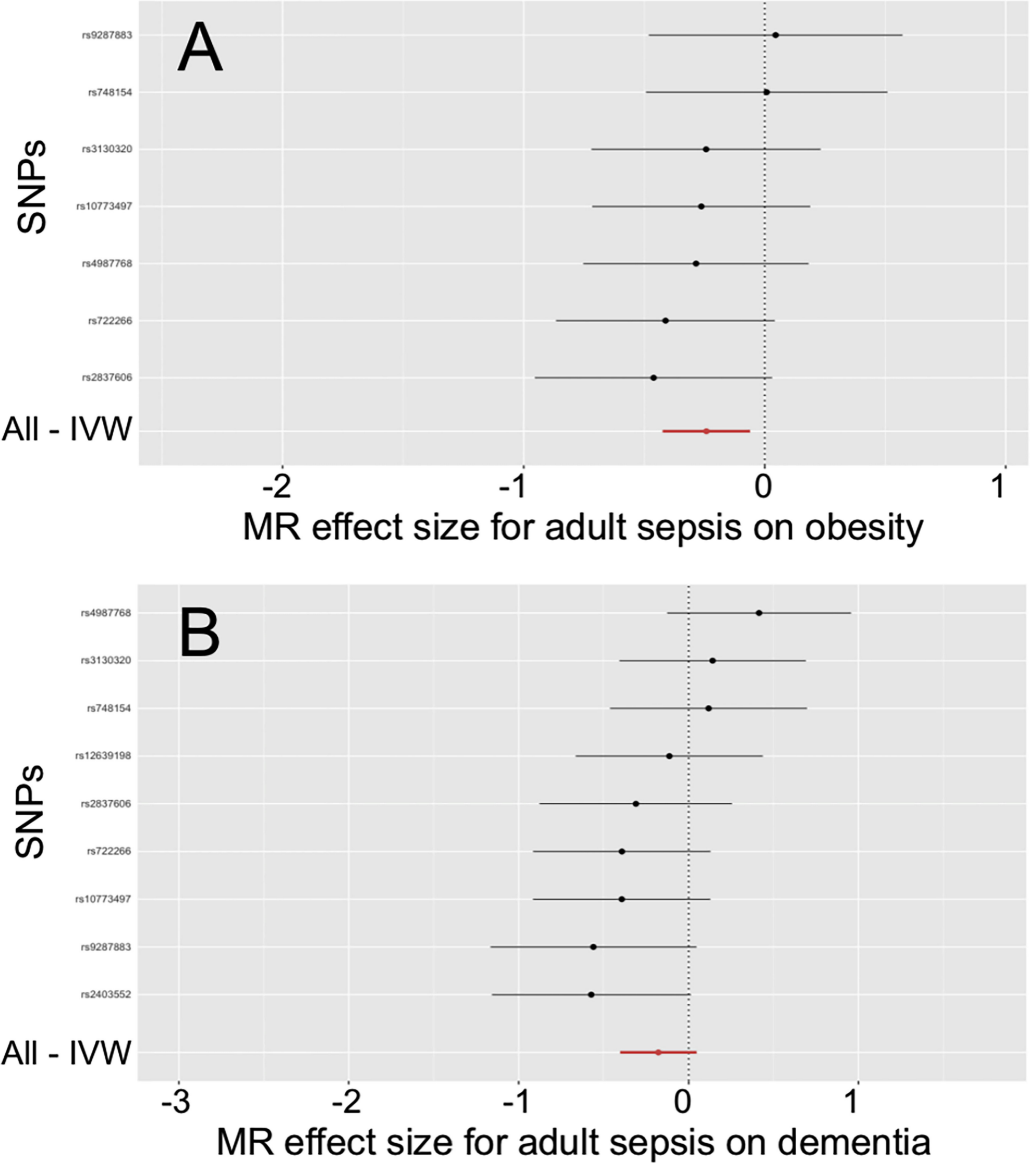
Effects of adult sepsis on obesity and dementia. **(A)** Forest plot for the effect of adult sepsis on obesity. **(B)** Forest plot for the effect of adult sepsis on dementia. SNP, Single nucleotide polymorphism; IVW, Inverse-variance weighted; MR, Mendelian randomization.

In **Table 3**, the IVW, MR-PRESSO and weighted median reported that adult sepsis was significantly related to the decreased risk of obesity (OR = 0.785, 95%CI = 0.655 ~ 0.940, P = 0.009; OR = 0.785, 95%CI = 0.684 ~ 0.900, P = 0.013; OR = 0.768, 95%CI = 0.598 ~ 0.986, P = 0.038). The MR-Egger result was in the same direction as the IVW result, although it was still not statistically significant. In **Figure 2A** and **Supplemental Figures 10A, B**, the scatter plot and forest plot visualized the above results. In **Table 3** and **Supplemental Figures 10C, D**, the Cochran’s Q test failed to find any heterogeneity (P = 0.751), the MR-Egger intercept test (P = 0.576) and funnel plot proved that there was no horizontal pleiotropy in these analyses, and the MR-PRESSO test (P = 0.789) and leave-one-out test also failed to find any significant outliers in these analyses.

**Table 3:** MR results for adult sepsis affecting inflammation-related diseases in multiple physiological systems.

Outcomes/ Methods	P_MR ^a^	ORs	95% CIs	P_CQ ^a^	P_IN ^a^	P_PR ^a^
Asthma						
IVW	0.875	0.990	0.870 ∼ 1.126	0.229	0.822	0.241
MR-PRESSO	0.897	0.990	0.870 ∼ 1.126			
Weighted median	0.452	0.942	0.805 ∼ 1.101			
MR Egger	0.811	0.840	0.211 ∼ 3.343			
Allergy						
IVW	0.358	1.142	0.860 ∼ 1.516	0.303	0.261	0.333
MR-PRESSO	0.385	1.142	0.860 ∼ 1.516			
Weighted median	0.889	1.026	0.714 ∼ 1.476			
MR Egger	0.231	6.327	0.401 ∼ 99.731			
Rheumatoid arthritis						
IVW	0.243	0.879	0.708 ∼ 1.091	0.534	0.144	0.573
MR-PRESSO	0.251	0.879	0.720 ∼ 1.073			
Weighted median	0.576	0.920	0.685 ∼ 1.234			
MR Egger	0.133	0.028	0.001 ∼ 1.395			
Obesity						
**IVW**	**0.009**	**0.785**	**0.655 ∼ 0.940**	0.751	0.576	0.789
**MR-PRESSO**	**0.013**	**0.785**	**0.684 ∼ 0.900**			
**Weighted median**	**0.038**	**0.768**	**0.598 ∼ 0.986**			
MR Egger	0.421	0.472	0.088 ∼ 2.527			
Type 2 diabetes						
IVW	0.098	1.094	0.983 ∼ 1.218	0.632	0.577	0.646
MR-PRESSO	0.097	1.094	0.998 ∼ 1.200			
Weighted median	0.412	1.061	0.921 ∼ 1.221			
MR Egger	0.696	0.810	0.296 ∼ 2.217			
Dementia						
IVW	0.120	0.837	0.668 ∼ 1.048	0.163	0.721	0.199
MR-PRESSO	0.159	0.837	0.668 ∼ 1.048			
**Weighted median**	**0.038**	**0.756**	**0.580 ∼ 0.985**			
MR Egger	0.622	0.534	0.050 ∼ 5.764			

^a^ “P_MR” indicated “P value for Mendelian randomization”; “P_CQ” indicated “P value for Cochran’s Q test”; “P_IN” indicated “MR-Egger intercept test”; “P_PR” indicated “MR-PRESSO for horizontal pleiotropy”. Bold font indicates that the results are statistically significant (P<0.05).

The weighted median reported that adult sepsis was significantly associated with the decreased risk of dementia (OR = 0.756, 95%CI = 0.580 ~ 0.985, P = 0.038). However, the other three methods (including the IVW) did not report statistically significant results. In addition, the study did not find any association of adult sepsis with the other inflammation-related diseases. These results were listed in **Table 3**. The scatter plots, forest plots, funnel plots and leave-one-out tests for all results in this section were showed in **Figure 2** and **Supplementary Figures 7-12**.

## 4 Discussion

Currently, there were very little GWAS data on sepsis all around the world both in the adult and neonatal populations. However, as a critical infectious and inflammatory disease, sepsis may have a significant detrimental effect on a wide range of physiological systems, which must to be studied. Regretfully, previous observational studies had been limited by their own inherent limitations and had only provided some results at the phenotypic level that were not particularly reliable. Therefore, MR analysis using available GWAS data to infer genetic or causal associations of sepsis with inflammation-related diseases in multiple physiological systems in different age populations was essential.

According to the results of this study, adult sepsis may reduce the risk of future obesity by more than 20%. Neonatal sepsis also had the ability to reduce future body mass index levels, and the change was statistically significant. These results were unexpected, but were confirmed in both the adult and neonatal populations. It was important to note that the two results were based on completely different GWASs, which made these results seem more credible. Based on the existing knowledge, we provided two potential explanations for these results. However, it was important to note that these mechanisms were only plausible speculations and must be verified by further studies.

**Figure 3:**
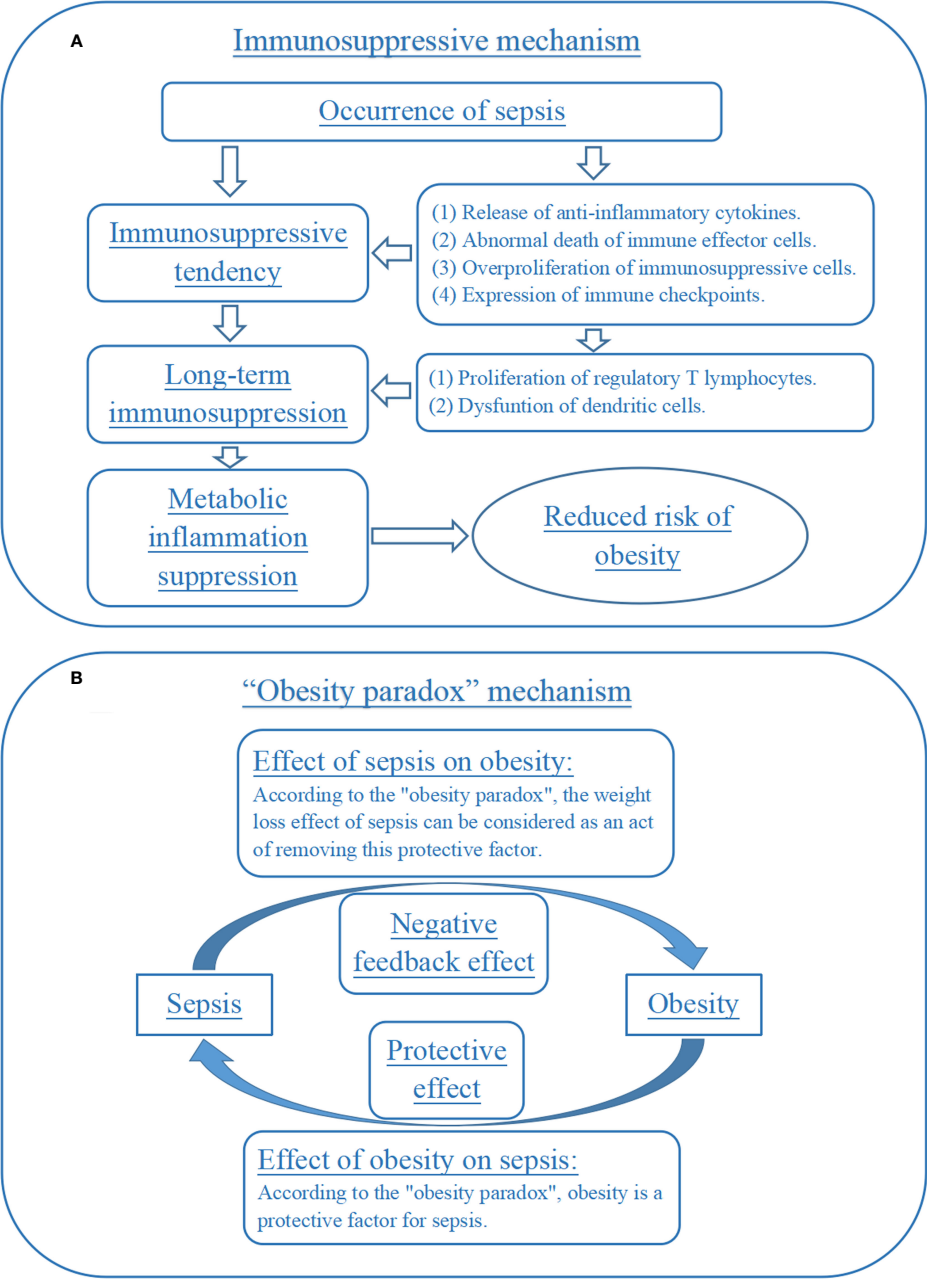
Immunosuppression and “obesity paradox” mechanisms for the effect of sepsis on obesity. **(A)** Immunosuppressive mechanism for the effect of sepsis on obesity. **(B)** “Obesity paradox” mechanism for the effect of sepsis on obesity.

The first explanation was tentatively named as immunosuppressive mechanism in this study (**Figure 3A**). Briefly, sepsis triggered a series of complex immune responses, which included both pro-inflammatory and anti-inflammatory mechanisms. Then, most sepsis patients may develop a significant tendency towards immunosuppression with the progression of the disease, caused by the release of anti-inflammatory cytokines, abnormal death of immune effector cells, overproliferation of immunosuppressive cells and expression of immune checkpoints (26, 27). More importantly, sepsis-induced immunosuppression may be a long-term process in many patients and was not alleviated with the disappearance of sepsis symptoms, which can be caused by the proliferation of regulatory T lymphocytes and dysfuntion of dendritic cells (28, 29). Meanwhile, obesity was considered to be an inflammatory disorder. A growing number of studies had found that anti-inflammatory measures may play an important role in reducing the risk of obesity (30, 31). So, the long-term immunosuppression caused by sepsis may be a potential mechanism for the reduction of body mass index level and obesity risk in neonatal and adult sepsis in this study.

The second explanation can be named the “obesity paradox” mechanism (**Figure 3B**). Briefly, obesity had a negative impact on long-term health, but its effect on acute illnesses was complex. Some studies had proposed an “obesity paradox”, suggesting that in acute illnesses (such as sepsis), overweight and obese patients had a better prognosis than normal weight patients (32, 33). Some basic experiments had also explored the potential mechanisms included. For example, one animal experiment reported that obesity attenuated inflammation, protein catabolism, dyslipidaemia and muscle weakness during sepsis (34). If the “obesity paradox” was indeed valid, then the reduced risk of obesity exhibited by sepsis patients for a period of time after recovery may be seen as a “deleterious” effect or negative feedback effect of the disease on the patient’s health, which was trying to weaken the body’s protective processes against sepsis. This was an interesting but less rigorous speculation that should be verified by future research.

In addition, in the neonatal sepsis MR, the IVW and MR-PRESSO reported that the disease may reduce the risk of allergy, but the MR-Egger reported a result in the opposite direction. So, this analysis failed to meet the criteria for a positive result in this study. However, one observational study suggested that clinical sepsis in neonates might reduce the risk of developing allergic diseases in early childhood in children whose mothers have allergies (35). Another observational study mentioned above provided some similar results (15). Also, the mechanism underlying this potential relationship should also be related to immunosuppression following sepsis. Therefore, it was necessary to conduct further MR study to verify the genetic effect of sepsis on allergies when GWAS data were much updated.

The greatest advantage of the study was the MR strategy. It had a much larger sample size than previous observational studies, which led to higher test efficacy and more reliable results. Because it was a genetic level study, this MR study was less subject to epidemiological confounding factors. Also, the GWAS data it used were pre-adjusted for age, sex, etc., which further reduced the risk of confounding bias in this study. However, there were still some factors, such as socioeconomic status and lifestyle, that were not effectively adjusted for in the population of this study, which may affect the reliability of the results in this study. In addition, the MR study explored causal correlations at the genetic level, so its results were highly unlikely to be affected by reverse causation. However, this was still an issue that cannot be completely ignored, because the factors such as obesity (the outcomes in this study) did have a potential impact on sepsis (the exposure in this study) in different age groups. So, these limitations should be well investigated in the future.

The selection of instrumental variables was a critical step in performing MR analysis. In this study, eleven and ten instrumental variables were selected from the above-mentioned GWASs for predicting neonatal and adult sepsis, respectively. Among them, the instrumental variables for adult sepsis fully satisfied the three major assumptions of MR and ensured the reliability of causal inference; while due to the lack of GWAS data, the instrumental variables for neonatal sepsis were slightly less correlated with the exposure, which might have some impact on MR results. However, we still considered the process and results of the study to be acceptable. The reasons were as follows: Firstly, previous studies had used instrumental variables with similar levels of correlation to neonatal MR and had achieved meaningful results (23). Secondly, the instrumental variables in adult MR had instrumental variables with better genome-wide correlation, while the results of adult and neonatal MR regarding obesity can be corroborated with each other. Third, these MR results were mutually supportable with those from previous observational studies and basic experiments. More importantly, due to the urgency of sepsis prevention and control, it was essential to use the available data to conduct MR studies to obtain some valuable results. However, it was important to note that the results from the study should still be validated in the future.

In conclusion, this study found that sepsis had the potential to reduce the risk of obesity or body mass index level, both in neonates and in adults. This study also provided some inadequate evidence to prove that sepsis may have an impact on the risk of allergies in the neonatal population. In addition, this study did not find any association of neonatal or adult sepsis with asthma, rheumatoid arthritis, type 1/type 2 diabetes and intelligence/dementia. These findings may update the understanding about the effect of sepsis on several physiological systems both in neonates and adults.

## Data availability statement

The original contributions presented in the study are included in the article/**Supplementary Material**. Further inquiries can be directed to the corresponding authors.

## Author contributions

XT and LW conducted the study design. All authors performed the literature research, data acquisition/collation and data analysis. SL and QW prepared the manuscript. All authors contributed to the article and approved the submitted version.
