# Supplementary material for: Effect of neonatal and adult sepsis on inflammation-related diseases in multiple physiological systems: a Mendelian randomization study
Source: Front Endocrinol (Lausanne). 2023 Jul 20;14:1215751. doi: 10.3389/fendo.2023.1215751 (PMC10400313; doi:10.3389/fendo.2023.1215751)
Supplement: Supplementary file 1 [file DataSheet_1.docx]

**Supplemental Table 1 Characteristics of the instrumental variables in the study**

| **No.** | **SNP** | **Effect allele** | **Other allele** | **EAF value** | **Beta value** | **SE value** | **P value** |
| --- | --- | --- | --- | --- | --- | --- | --- |
| Neonatal sepsis |  |  |  |  |  |  |  |
| 1 | rs7225568 | T | C | 0.3998 | 0.6259 | 1.1454 | 3.29E-06 |
| 2 | rs7835808 | C | T | 0.6002 | 0.6152 | 1.1420 | 3.23E-06 |
| 3 | rs597695 | C | T | 0.4380 | -0.5621 | 1.1424 | 1.99E-05 |
| 4 | rs16877997 | A | G | 0.1024 | -0.9163 | 1.2402 | 1.96E-05 |
| 5 | rs12821366 | G | A | 0.1163 | 0.8920 | 1.2401 | 3.15E-05 |
| 6 | rs16832098 | G | A | 0.0917 | -1.2379 | 1.3338 | 2.44E-05 |
| 7 | rs10498688 | G | A | 0.2765 | -0.5978 | 1.1635 | 4.97E-05 |
| 8 | rs13228161 | C | G | 0.0946 | -0.9163 | 1.2506 | 4.55E-05 |
| 9 | rs1147855 | A | G | 0.2018 | 0.6627 | 1.1747 | 4.12E-05 |
| 10 | rs7094526 | G | A | 0.3882 | 0.5988 | 1.1589 | 4.37E-05 |
| 11 | rs1676959 | G | A | 0.2227 | 0.6471 | 1.1681 | 3.01E-05 |
| Adult sepsis |  |  |  |  |  |  |  |
| 1 | rs9287883 | A | G | 0.3112 | -0.0711 | 0.0153 | 3.40E-07 |
| 2 | rs12639198 | A | G | 0.2842 | 0.0758 | 0.0160 | 4.40E-07 |
| 3 | rs375309052 | A | G | 0.3301 | -0.0866 | 0.0196 | 8.00E-07 |
| 4 | rs3130320 | C | T | 0.6236 | -0.0771 | 0.0146 | 8.30E-10 |
| 5 | rs748154 | A | G | 0.3177 | 0.0755 | 0.0152 | 3.00E-07 |
| 6 | rs722266 | C | T | 0.8176 | -0.1002 | 0.0184 | 7.80E-08 |
| 7 | rs2403552 | T | C | 0.3289 | -0.0837 | 0.0181 | 4.20E-06 |
| 8 | rs10773497 | C | T | 0.4781 | 0.0729 | 0.0142 | 4.00E-07 |
| 9 | rs4987768 | A | C | 0.2569 | 0.0764 | 0.0163 | 4.60E-06 |
| 10 | rs2837606 | C | T | 0.6066 | 0.0689 | 0.0145 | 1.20E-06 |

Note: SNP = Single nucleotide polymorphisms; EAF = Effect allele frequency; SE = Standard error.


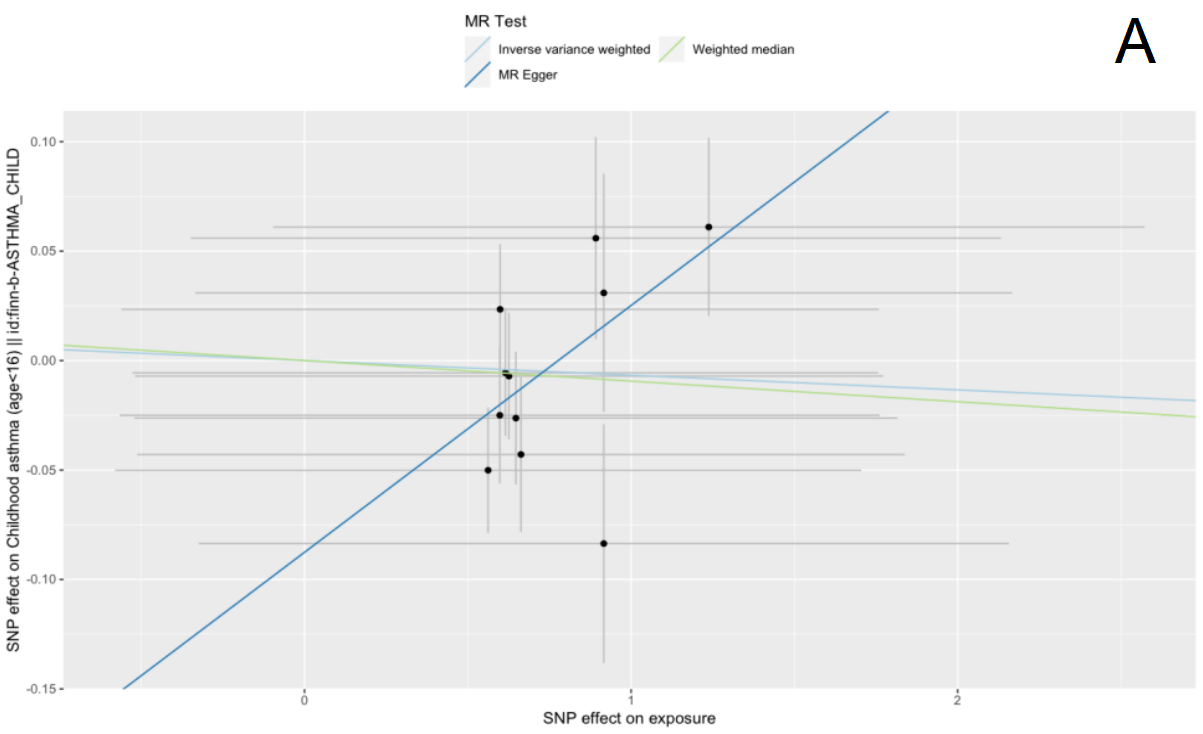

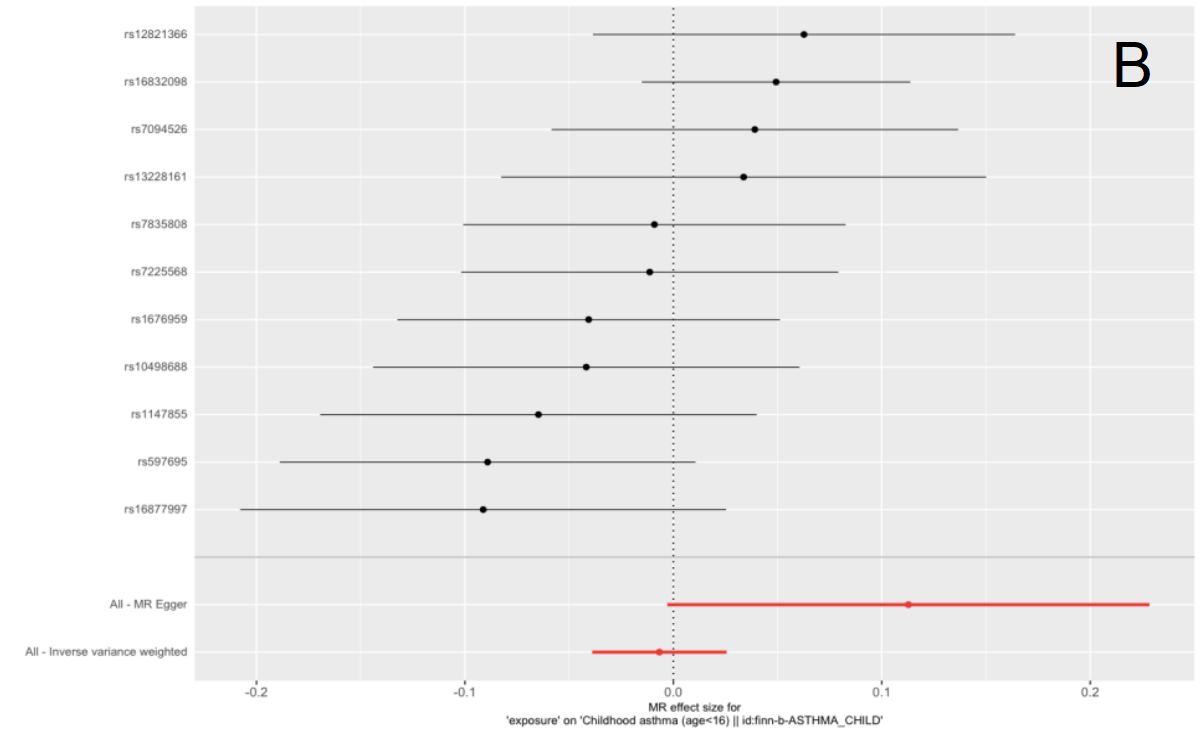


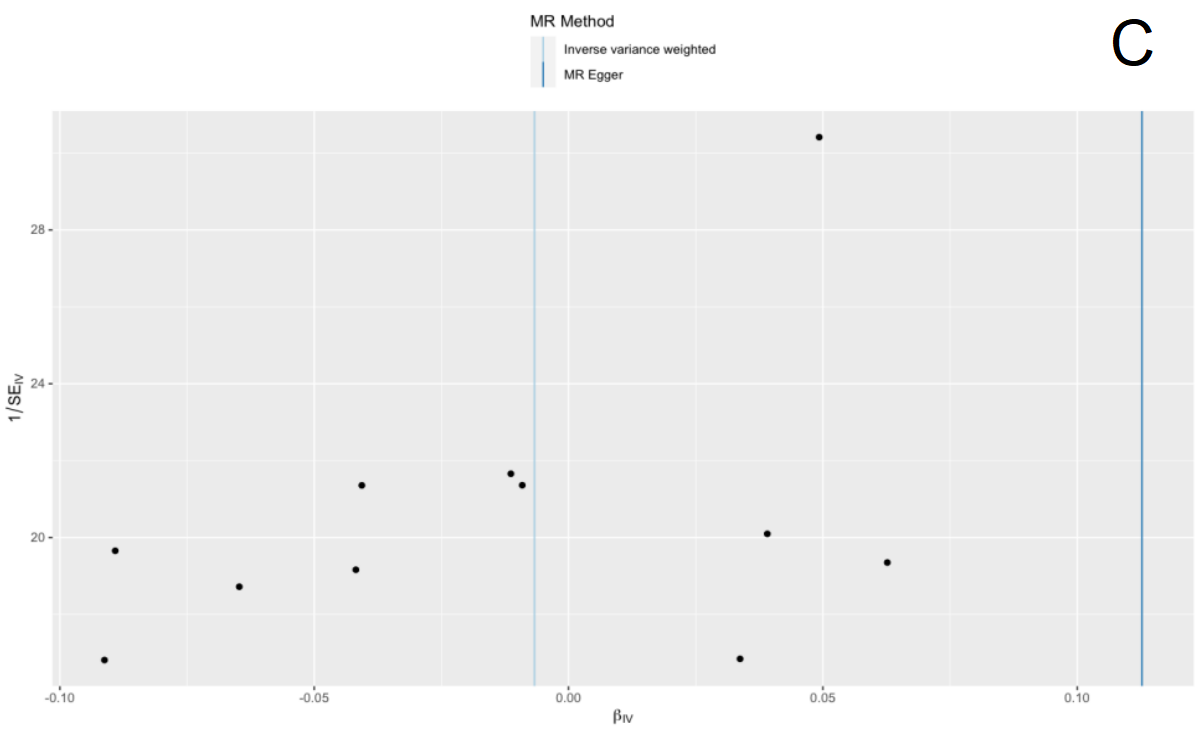

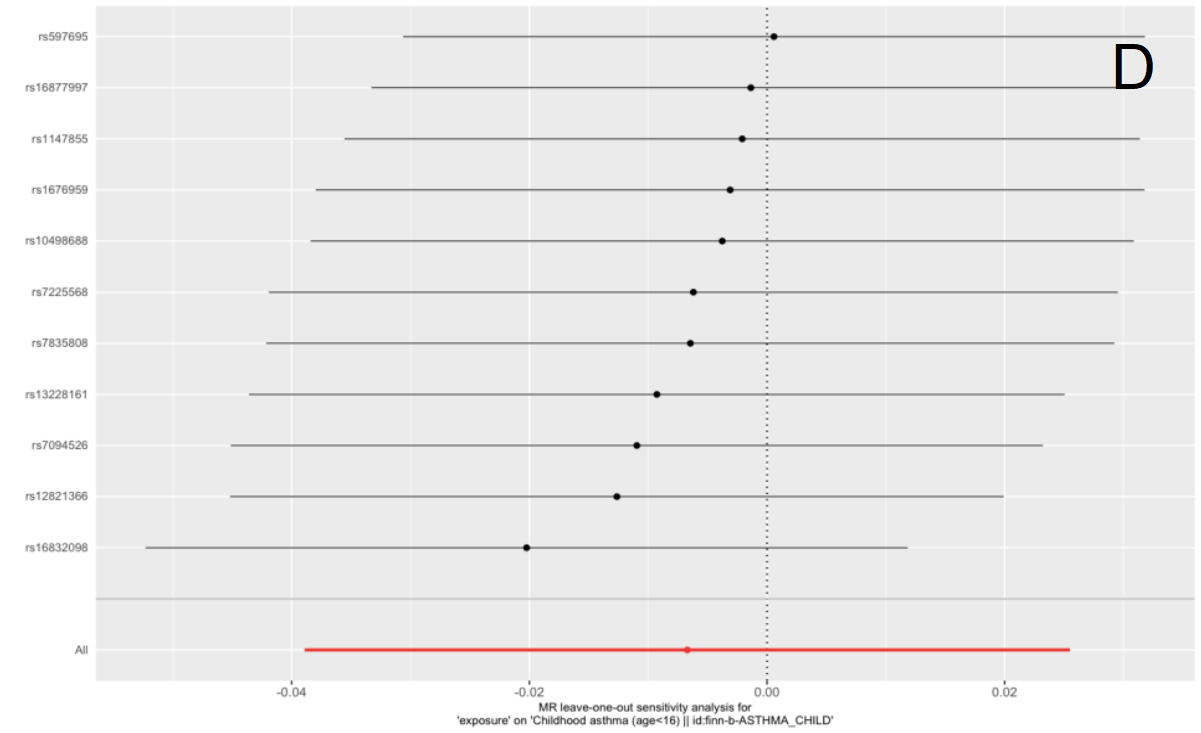


**Supplemental Figure 1 Scatter plot, forest plot, funnel plot and leave-one-out test for neonatal sepsis affecting asthma**

Figure 1A: Scatter plot; Figure 1B: Forest plot; Figure 1C: Funnel plot; Figure 1D: Leave-one-out test


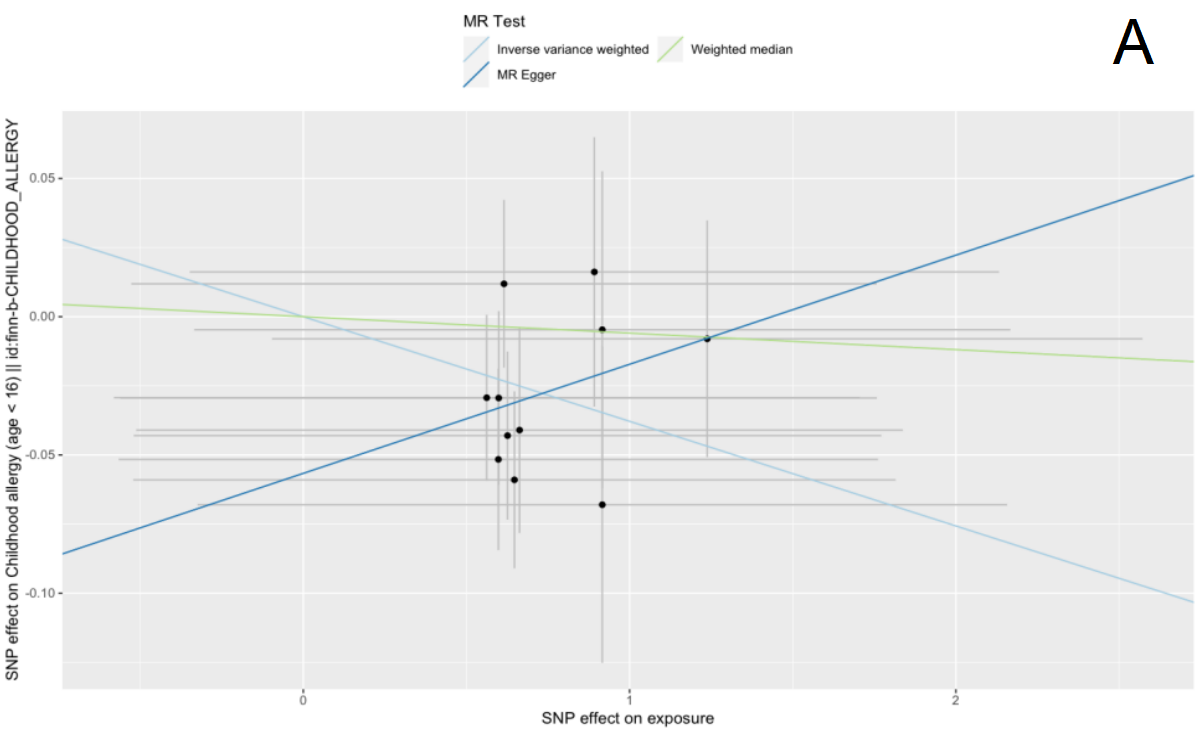

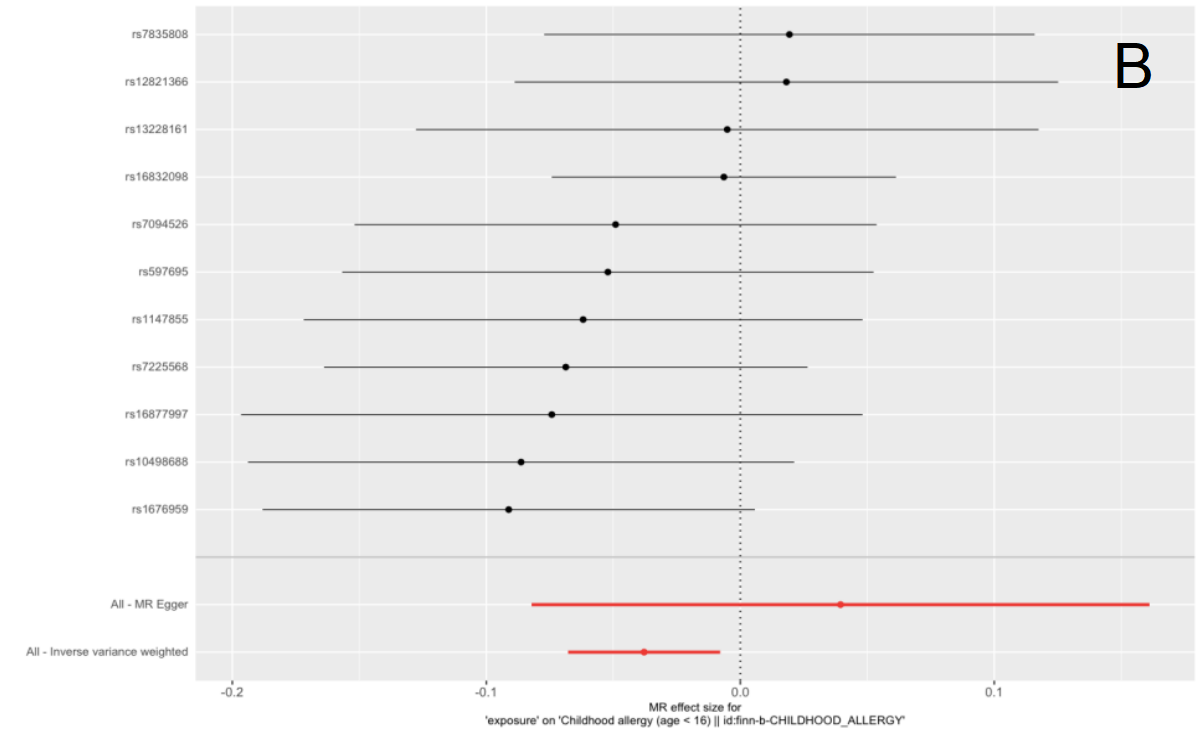


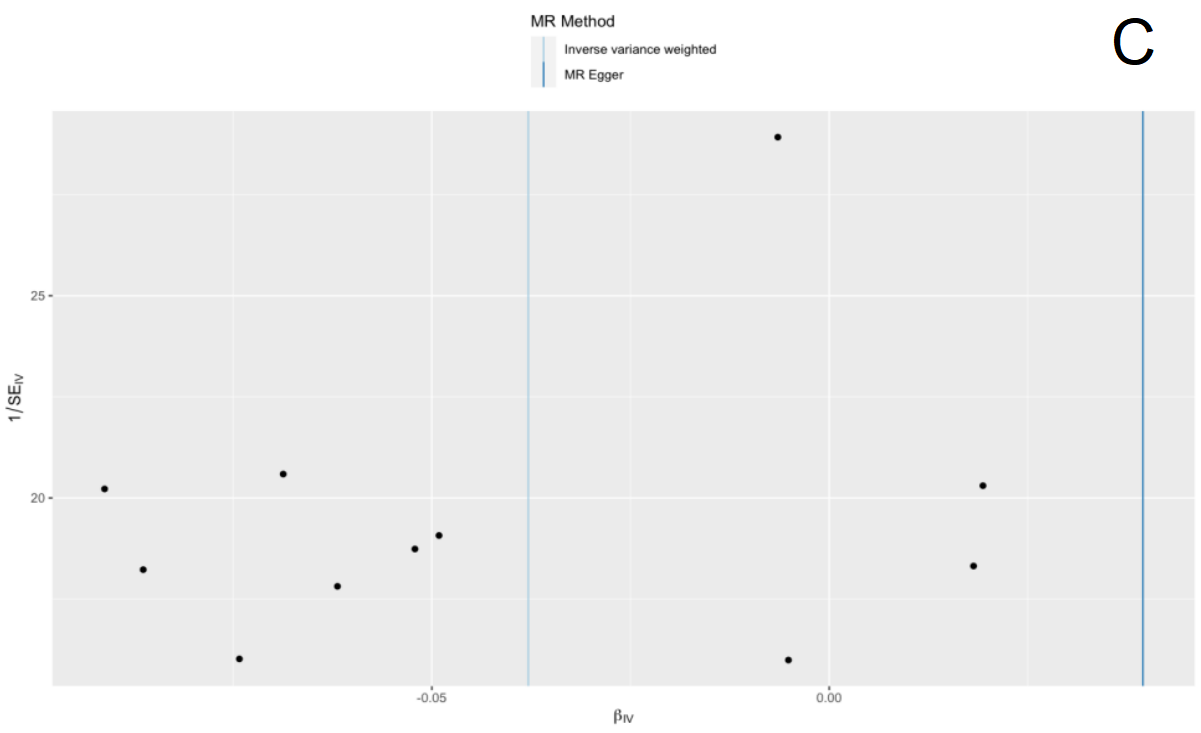

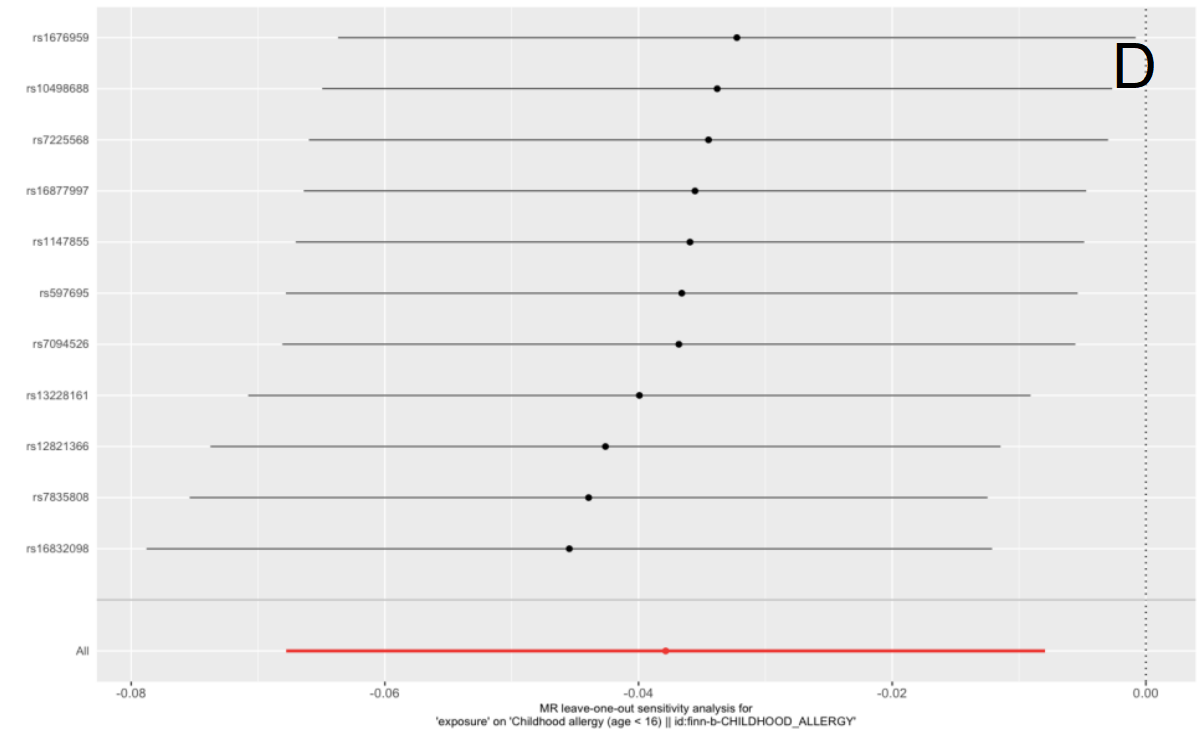


**Supplemental Figure 2 Scatter plot, forest plot, funnel plot and leave-one-out test for neonatal sepsis affecting allergy**

Figure 2A: Scatter plot; Figure 2B: Forest plot; Figure 2C: Funnel plot; Figure 2D: Leave-one-out test


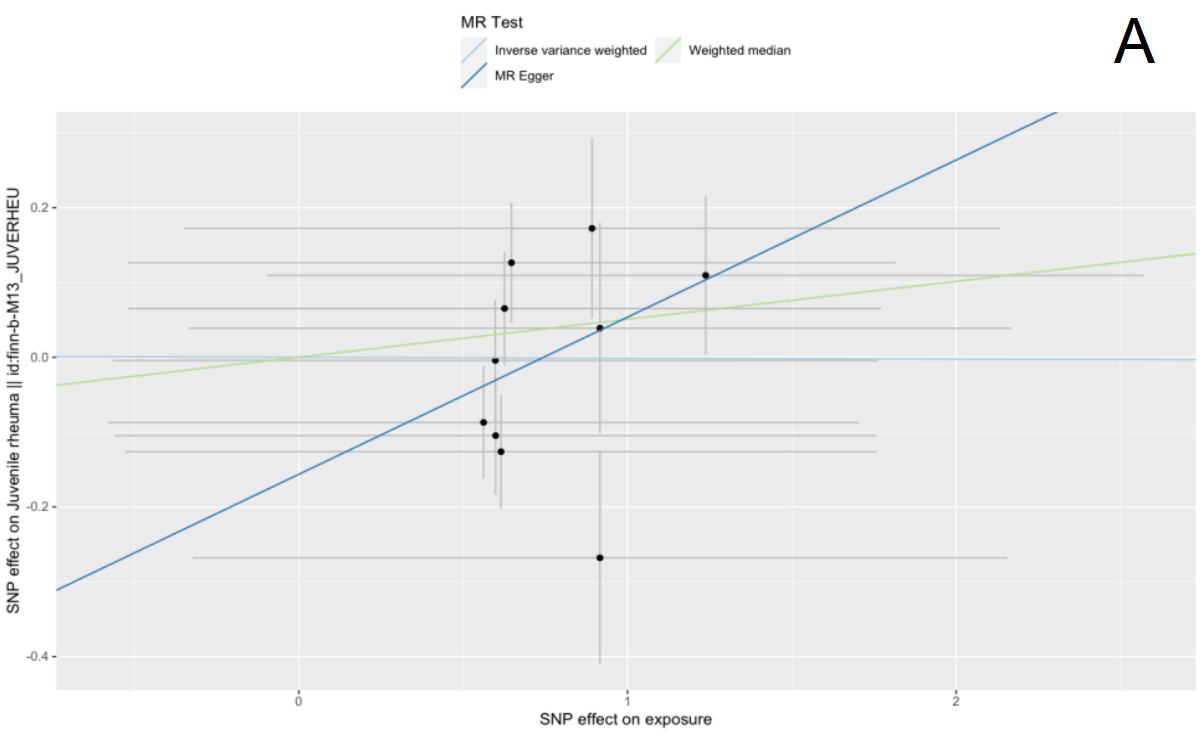

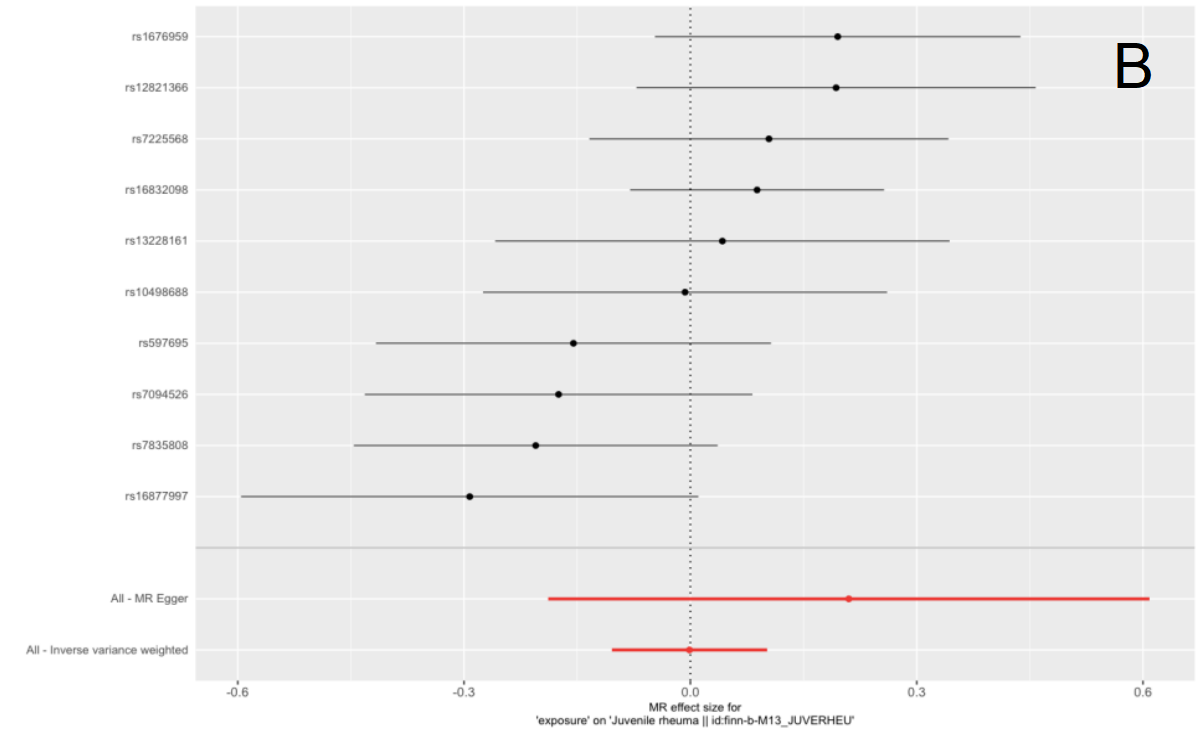


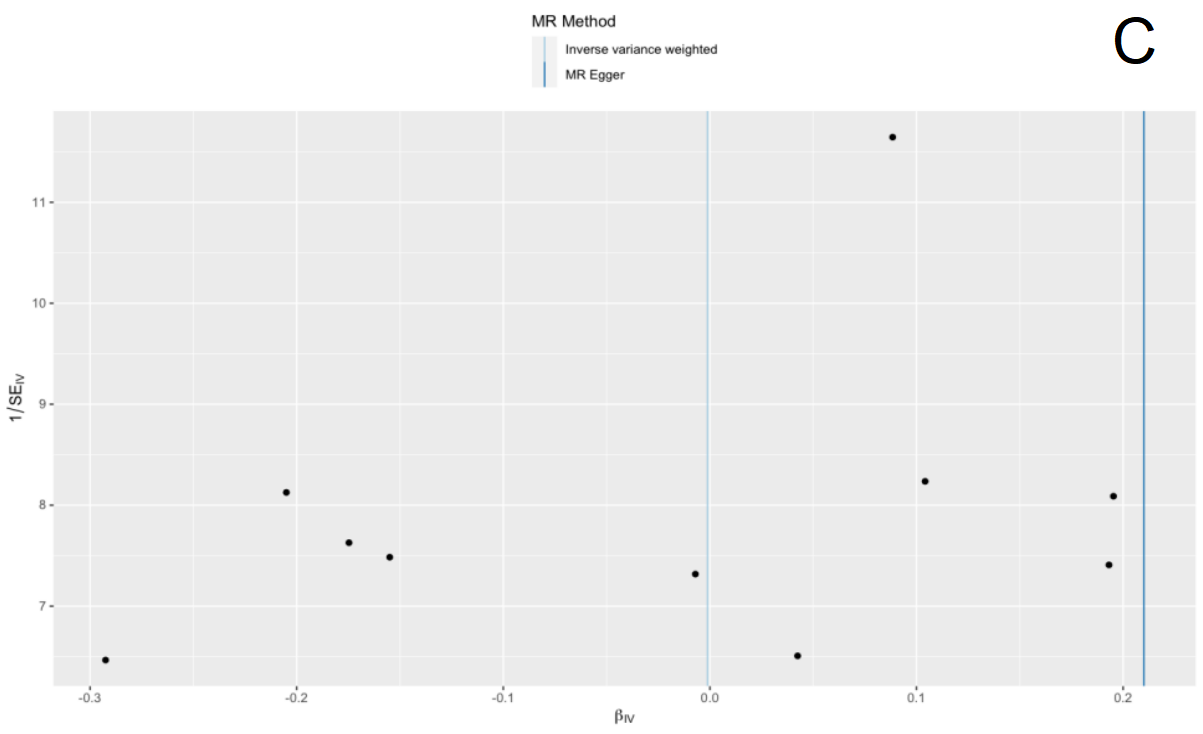

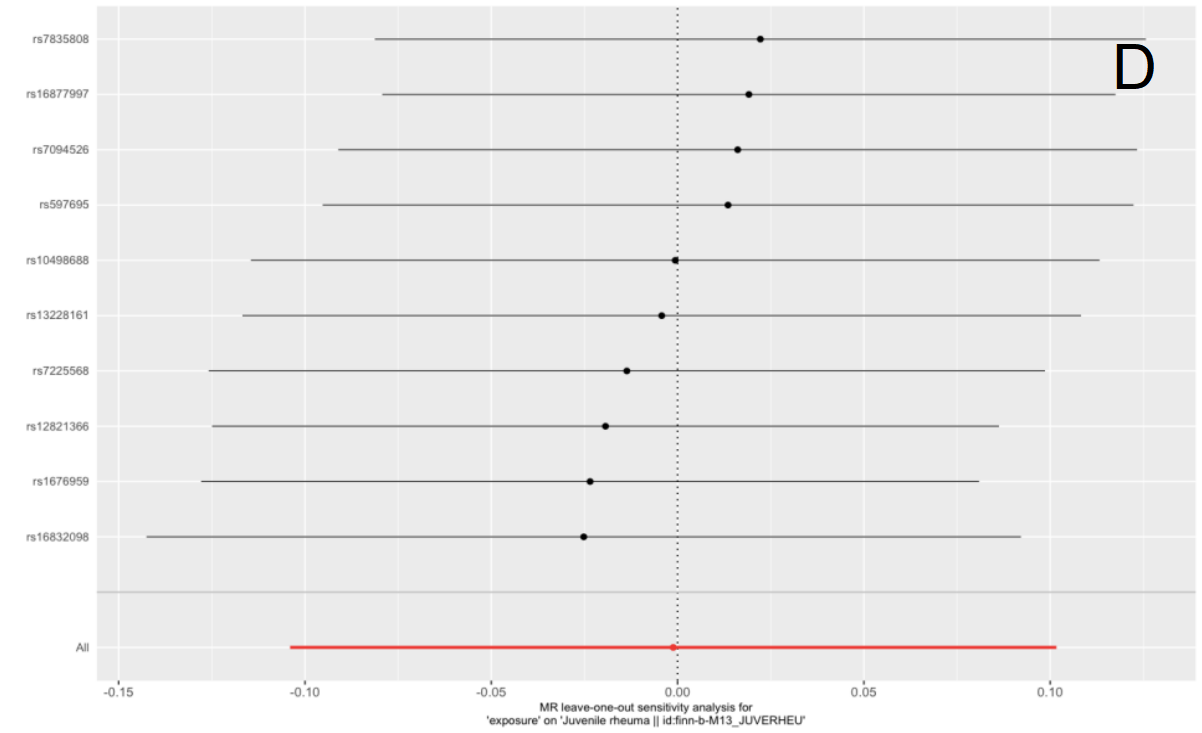


**Supplemental Figure 3 Scatter plot, forest plot, funnel plot and leave-one-out test for neonatal sepsis affecting juvenile rheuma**

Figure 3A: Scatter plot; Figure 3B: Forest plot; Figure 3C: Funnel plot; Figure 3D: Leave-one-out test


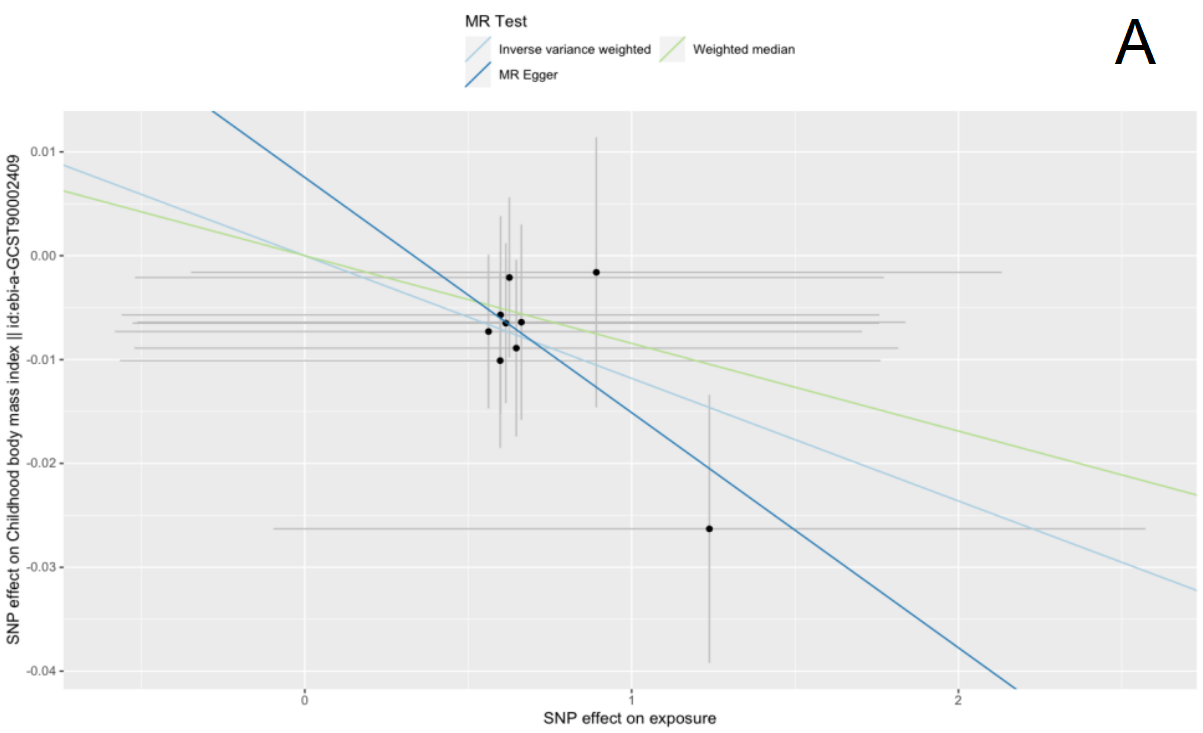

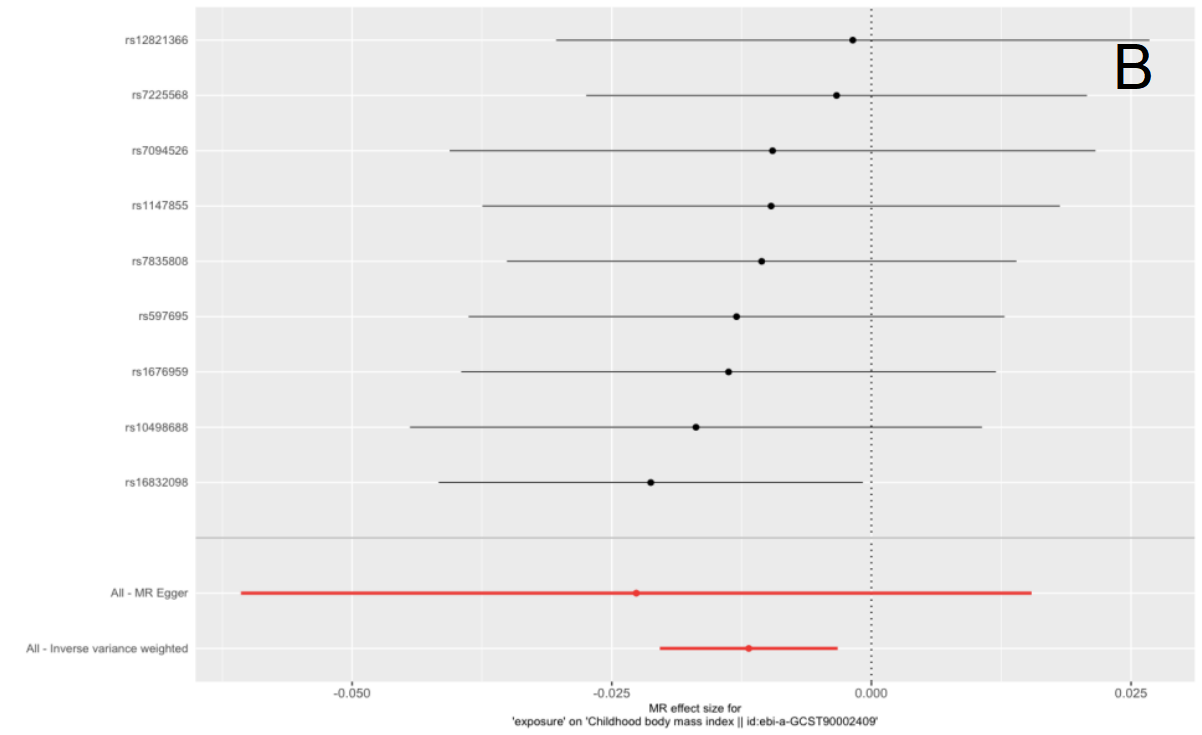


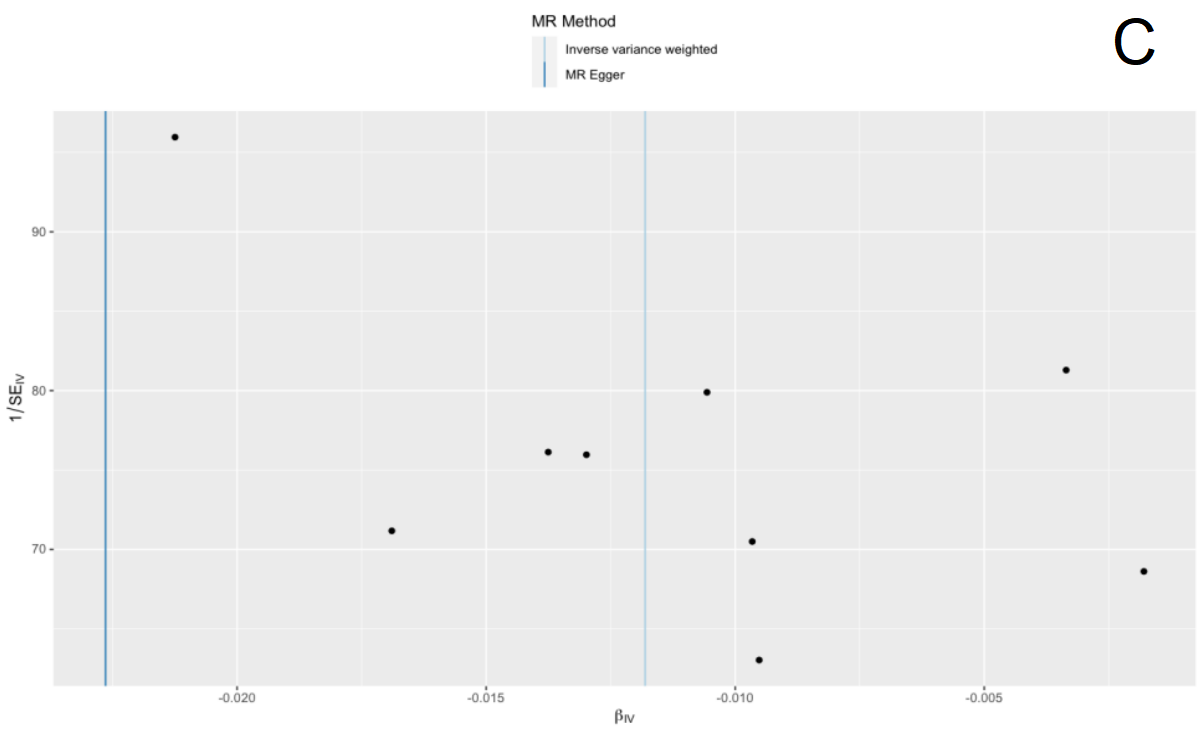

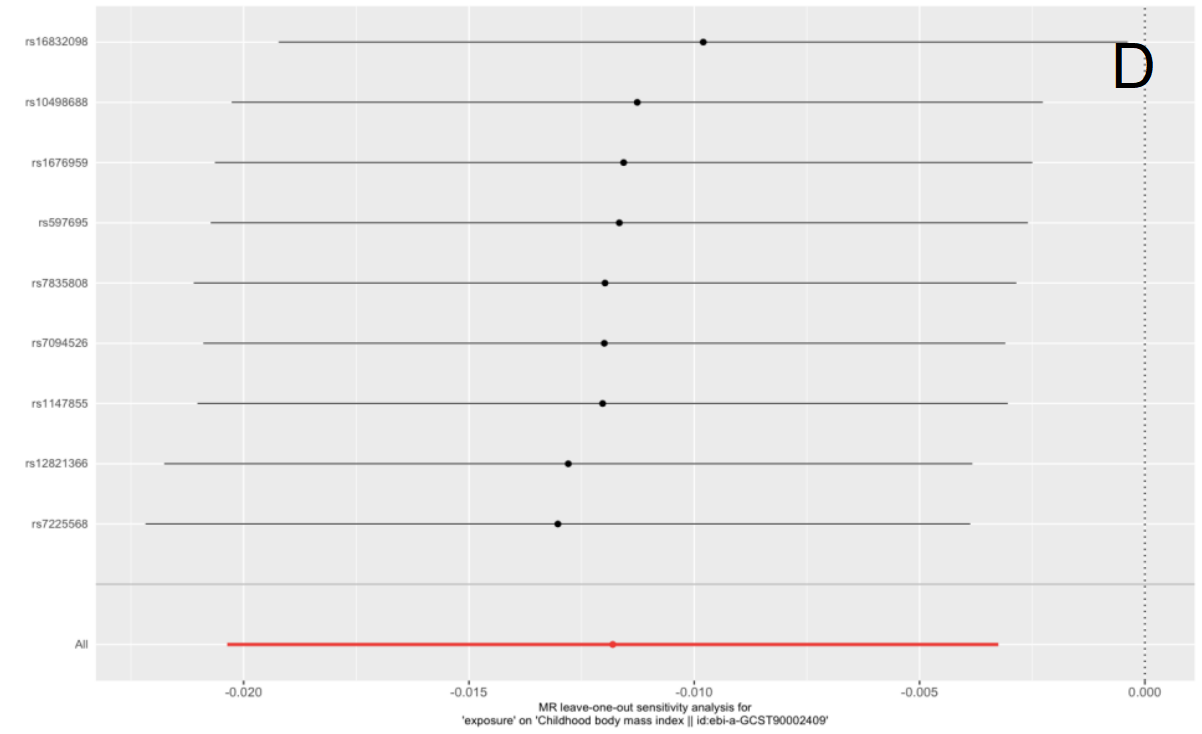


**Supplemental Figure 4 Scatter plot, forest plot, funnel plot and leave-one-out test for neonatal sepsis affecting body mass index**

Figure 4A: Scatter plot; Figure 4B: Forest plot; Figure 4C: Funnel plot; Figure 4D: Leave-one-out test


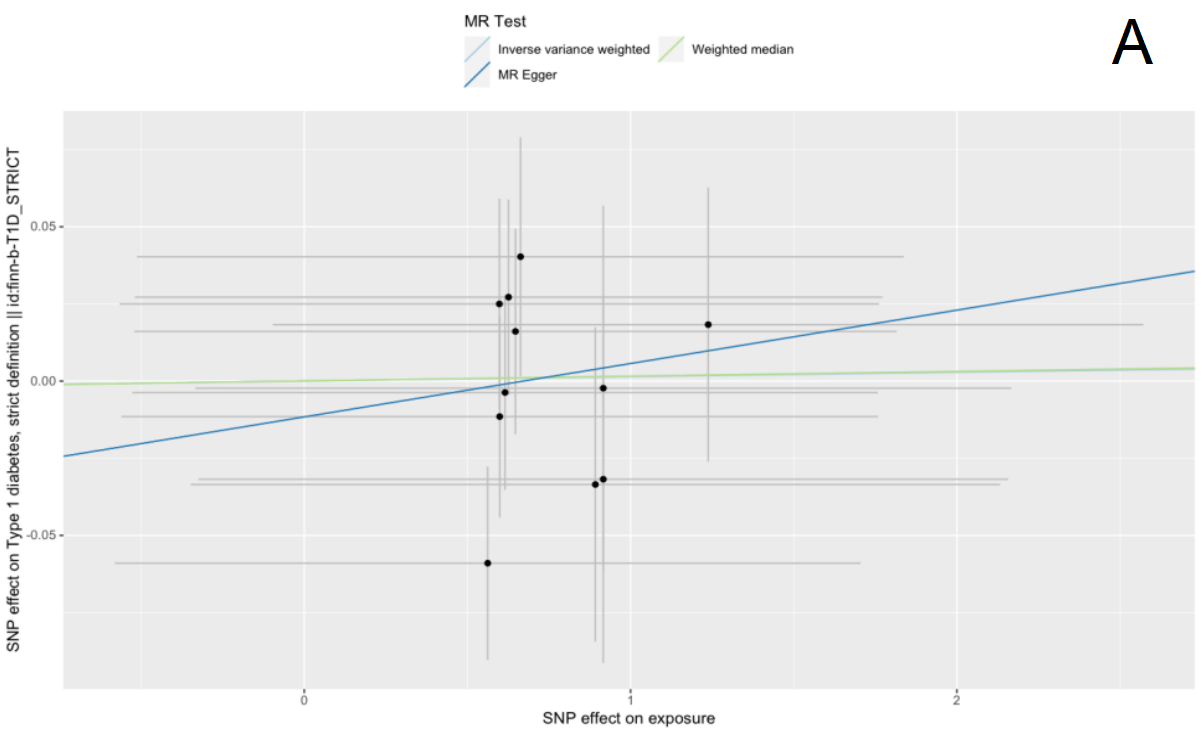

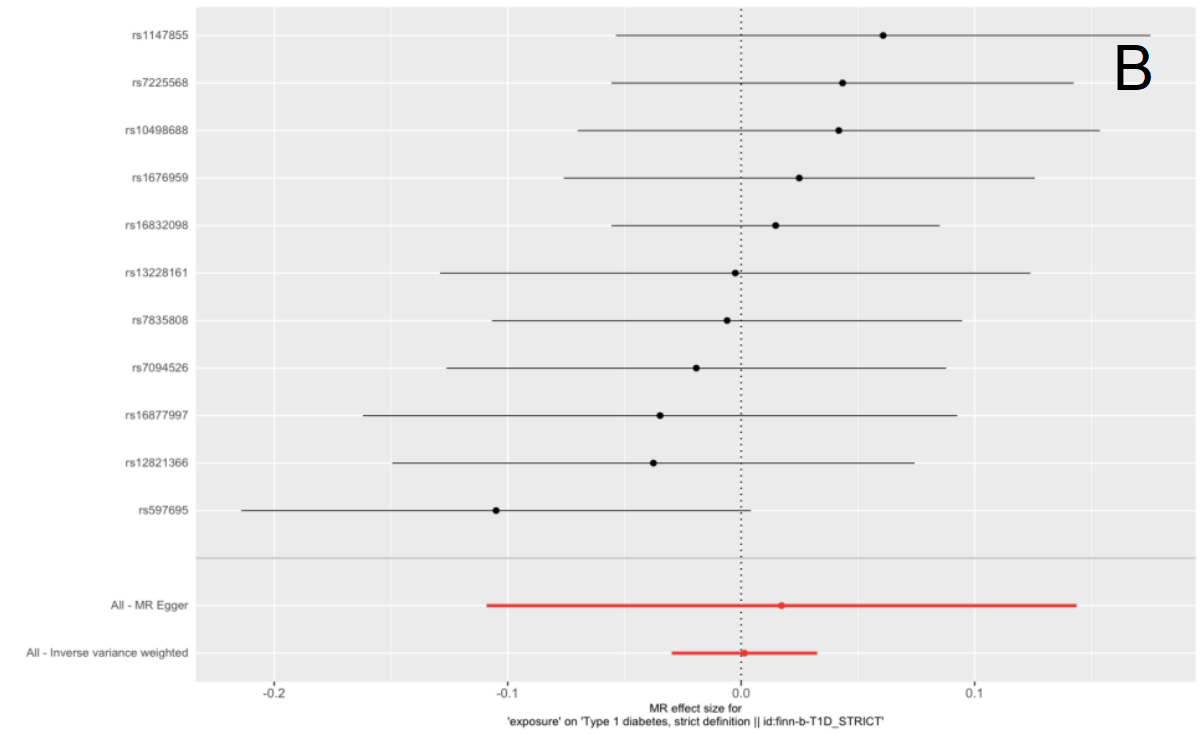


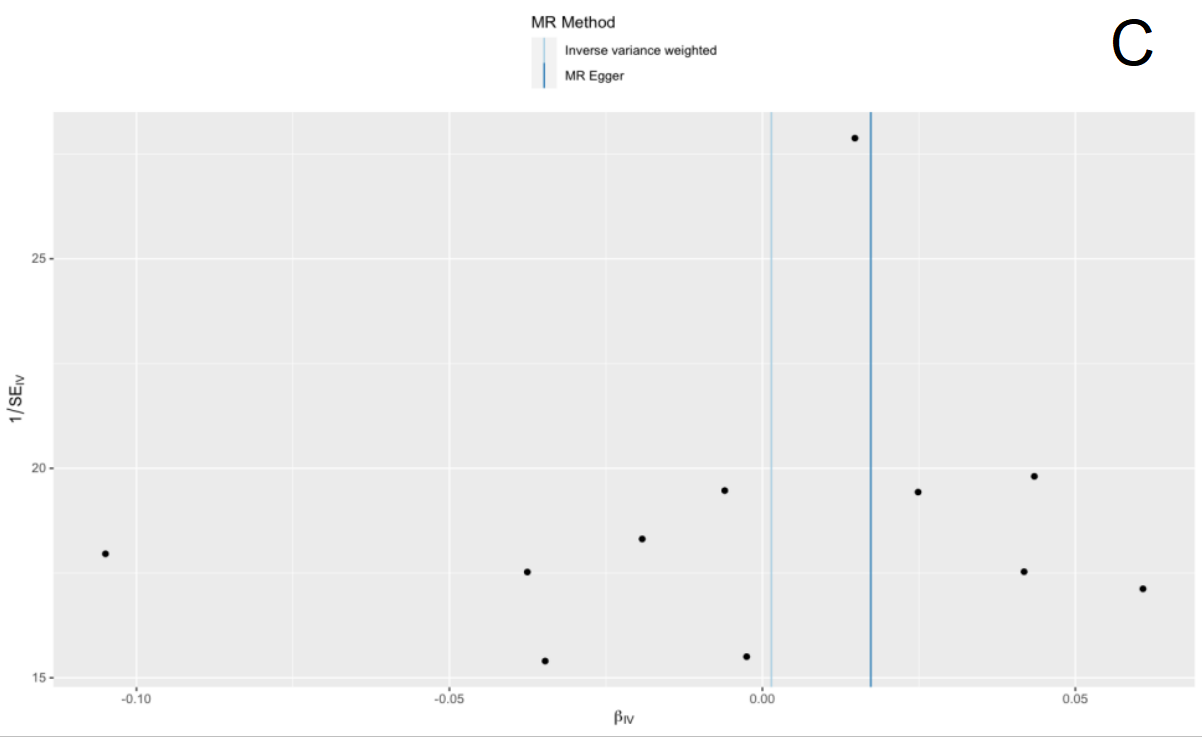

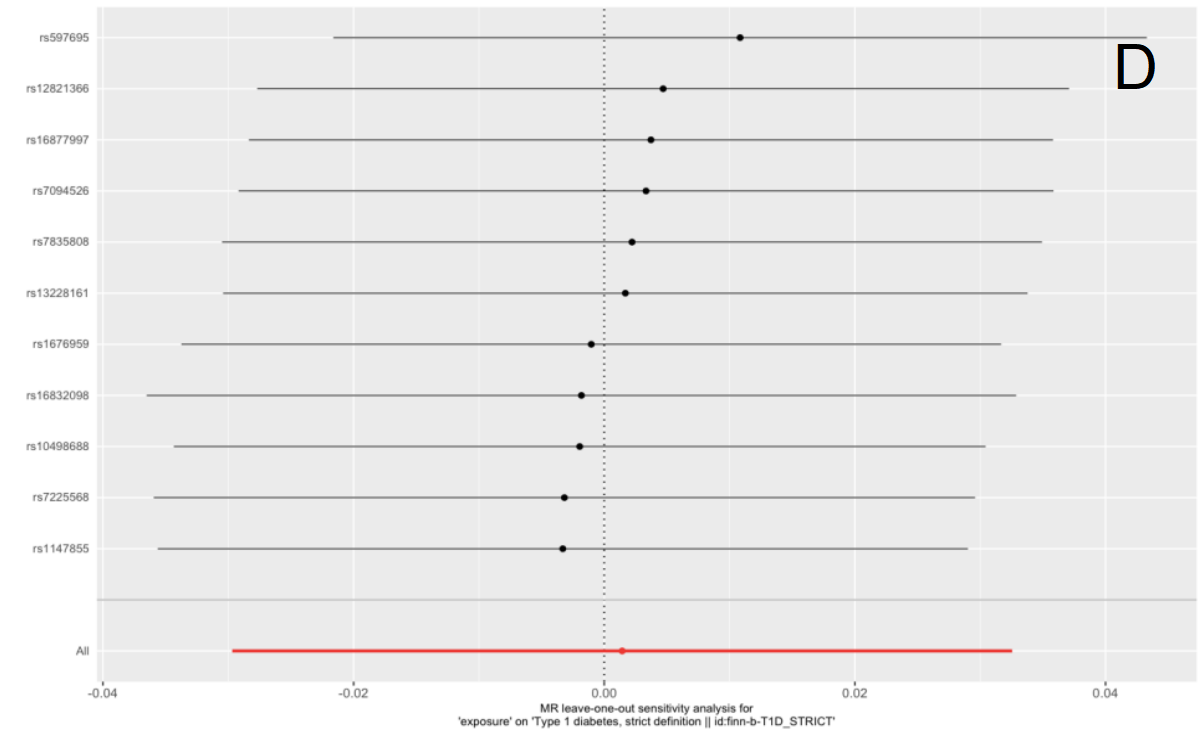


**Supplemental Figure 5 Scatter plot, forest plot, funnel plot and leave-one-out test for neonatal sepsis affecting type 1 diabetes**

Figure 5A: Scatter plot; Figure 5B: Forest plot; Figure 5C: Funnel plot; Figure 5D: Leave-one-out test


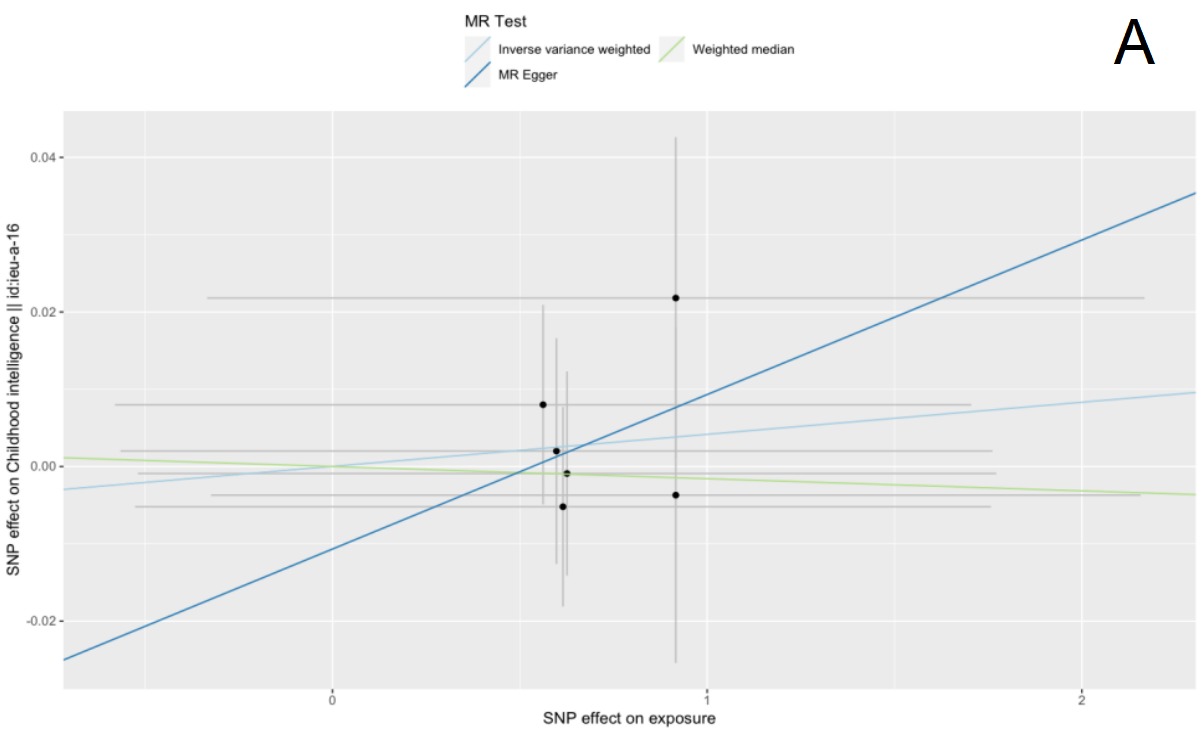

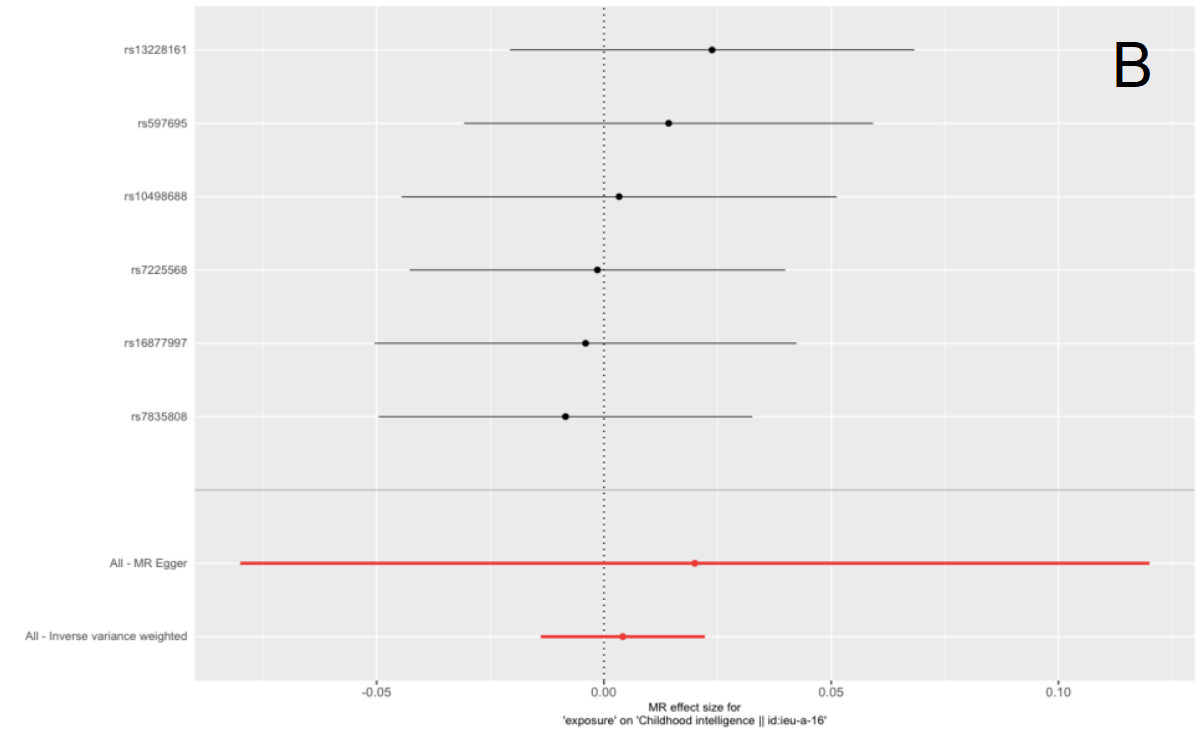


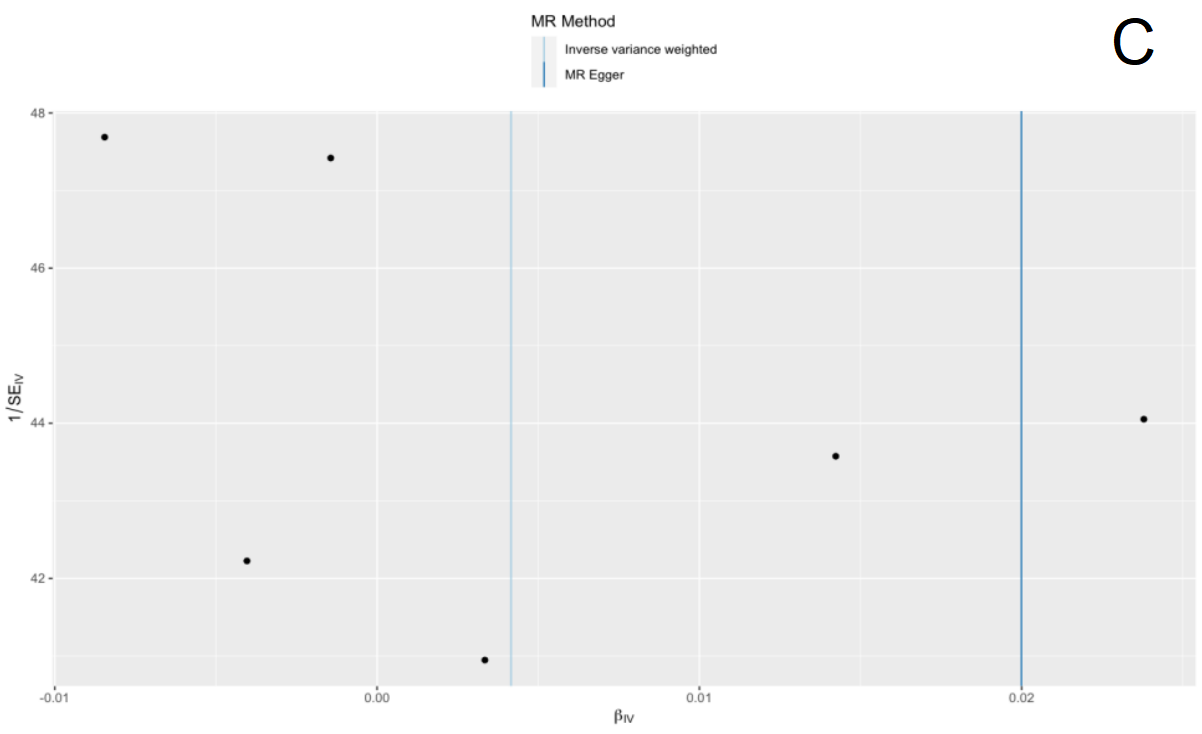

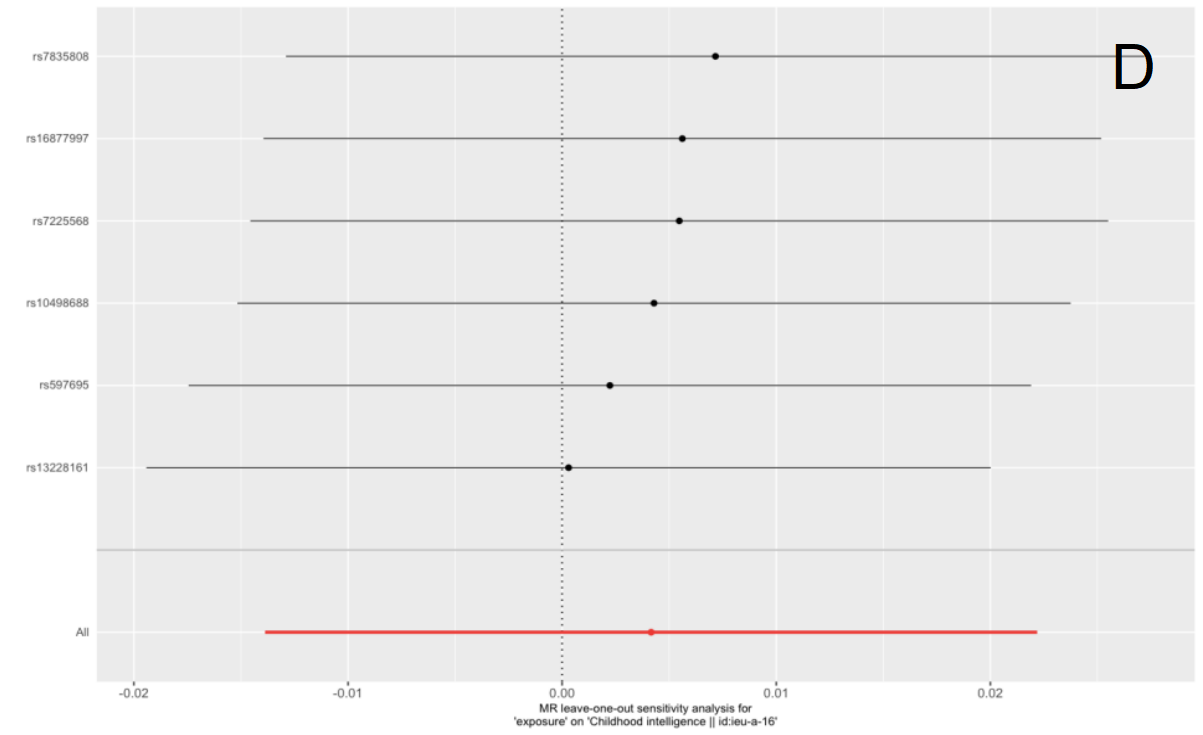


**Supplemental Figure 6 Scatter plot, forest plot, funnel plot and leave-one-out test for neonatal sepsis affecting intelligence**

Figure 6A: Scatter plot; Figure 6B: Forest plot; Figure 6C: Funnel plot; Figure 6D: Leave-one-out test


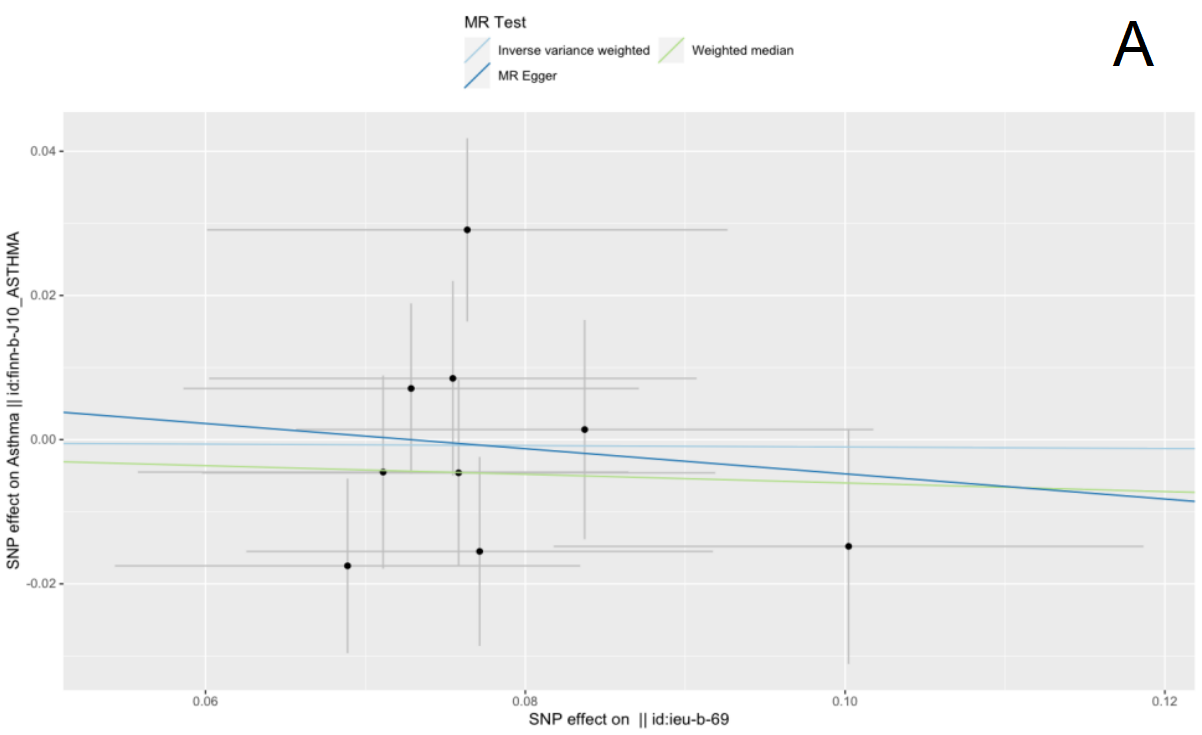

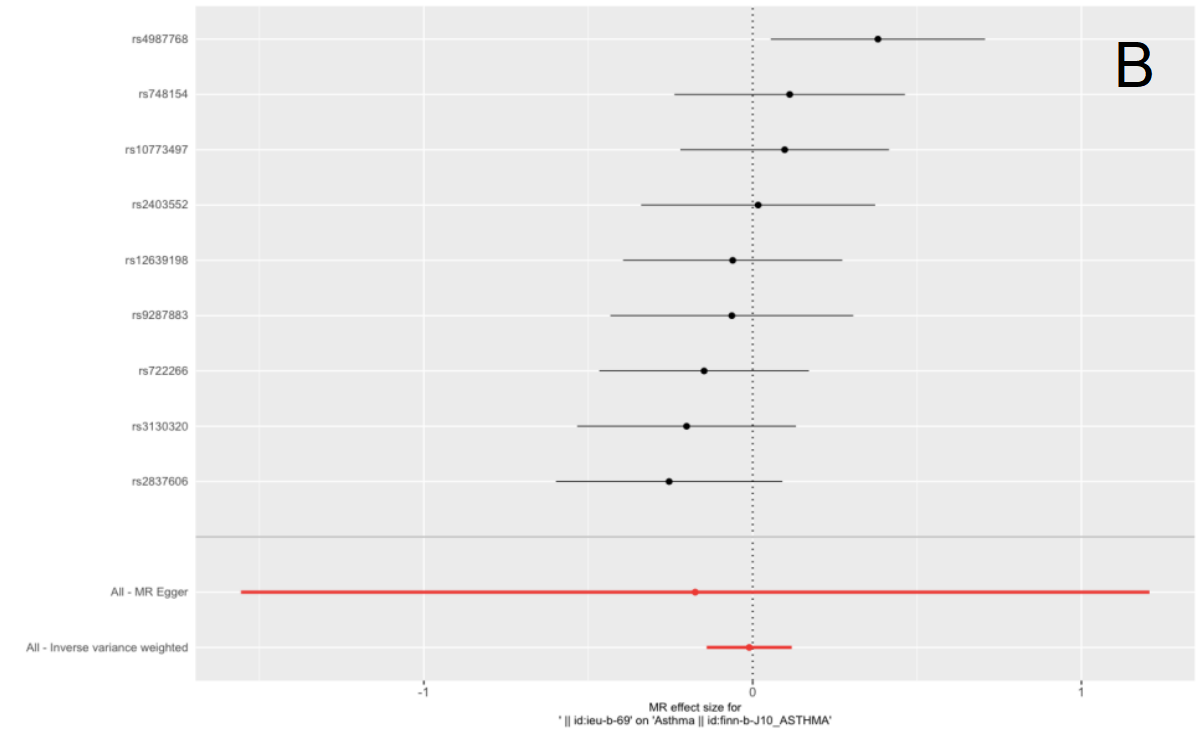


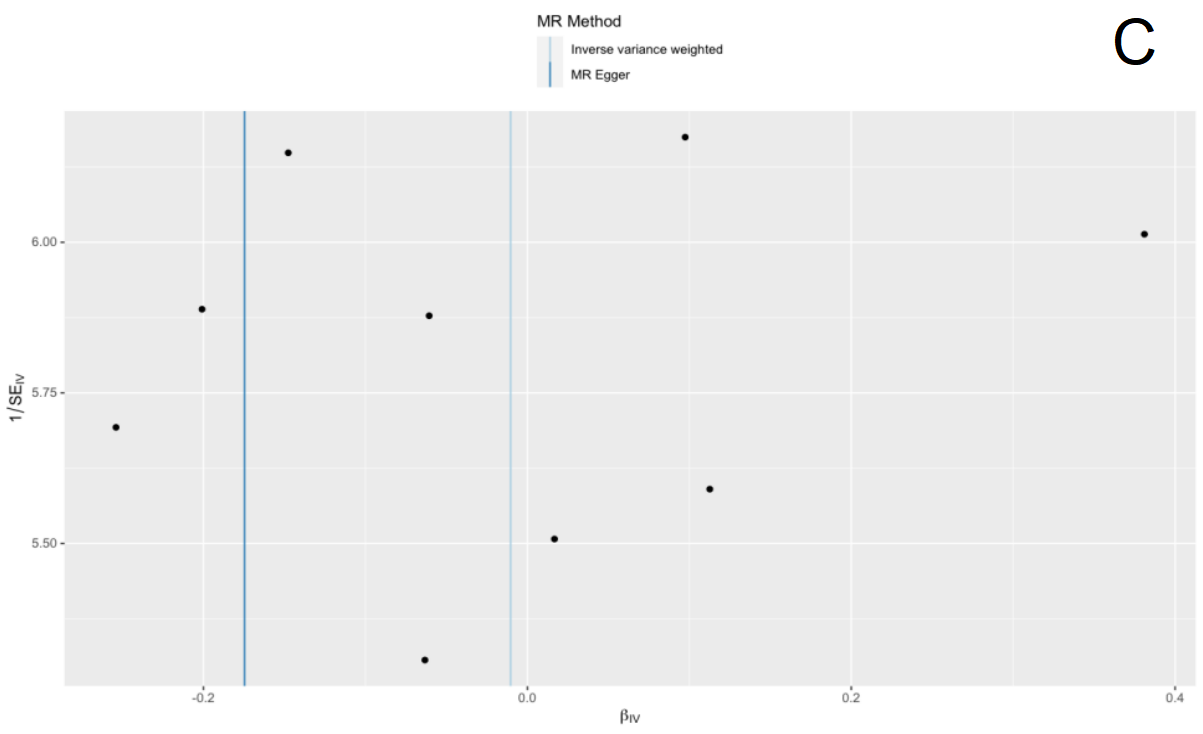

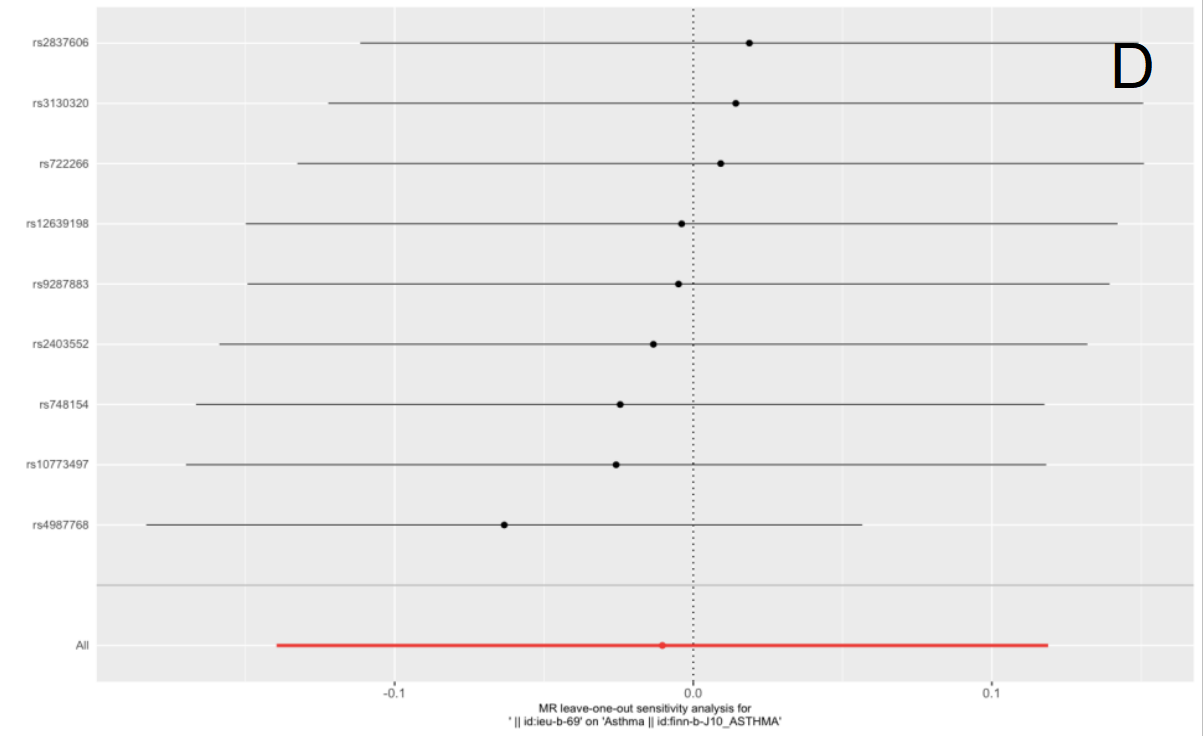


**Supplemental Figure 7 Scatter plot, forest plot, funnel plot and leave-one-out test for adult sepsis affecting asthma**

Figure 7A: Scatter plot; Figure 7B: Forest plot; Figure 7C: Funnel plot; Figure 7D: Leave-one-out test


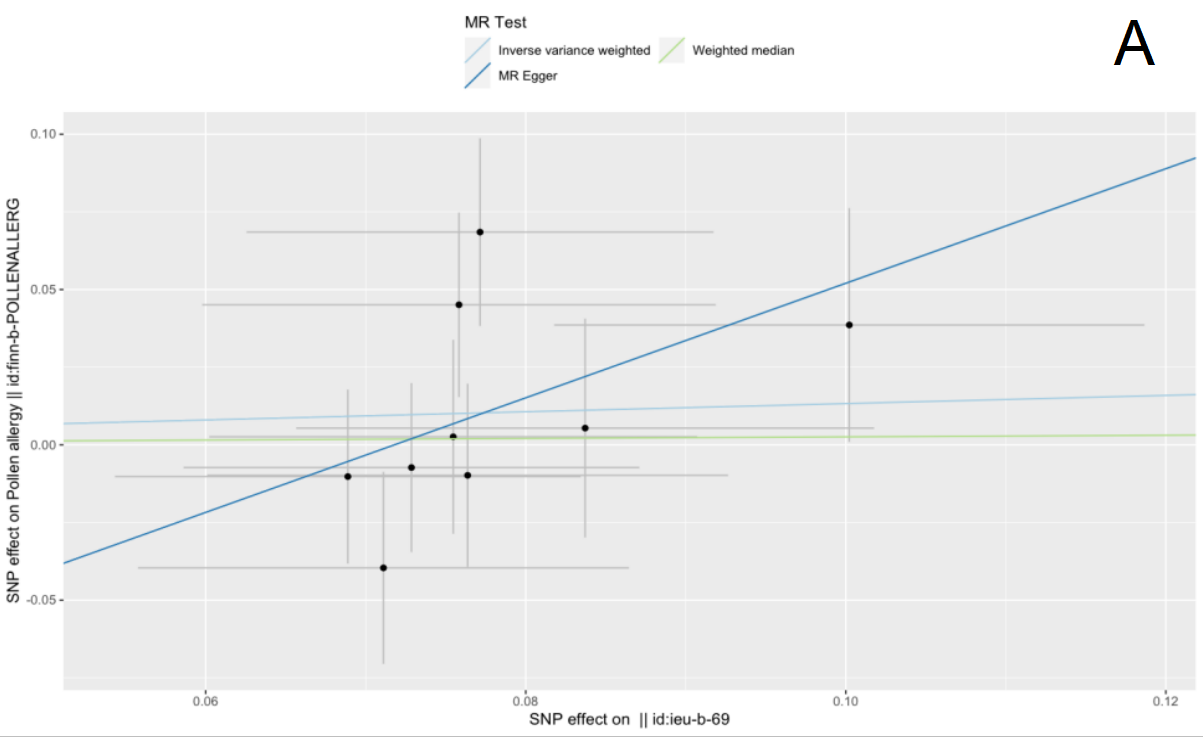

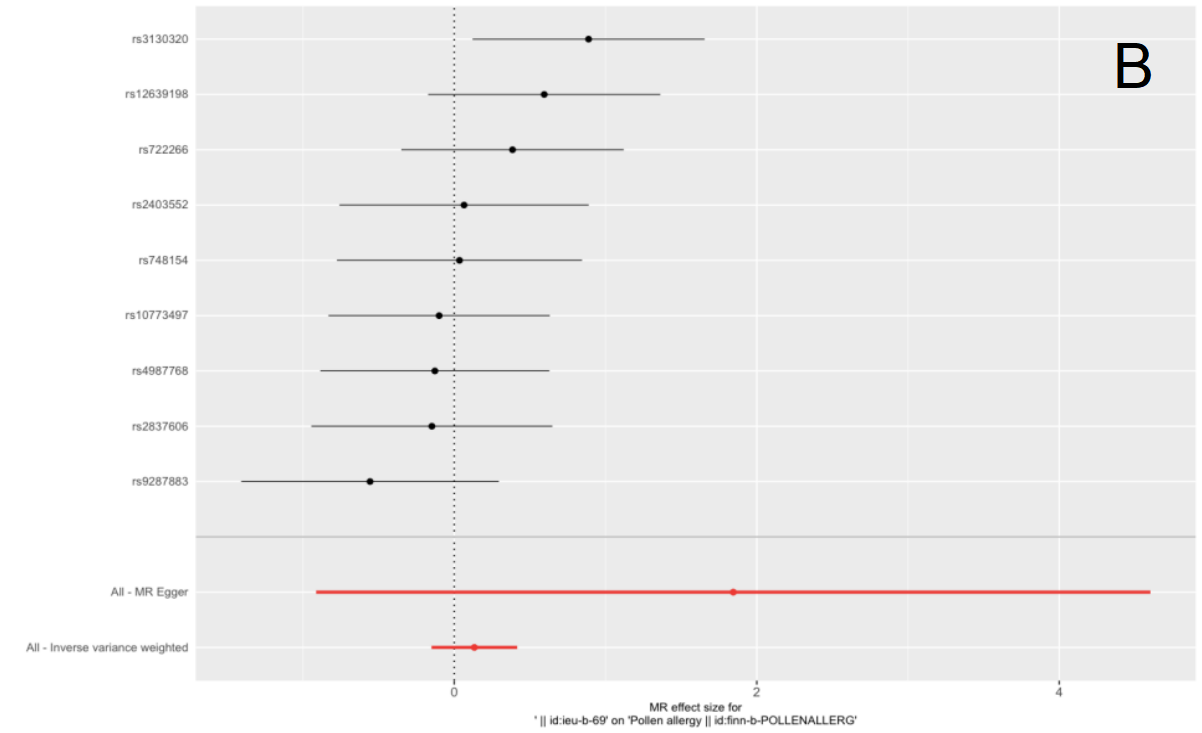


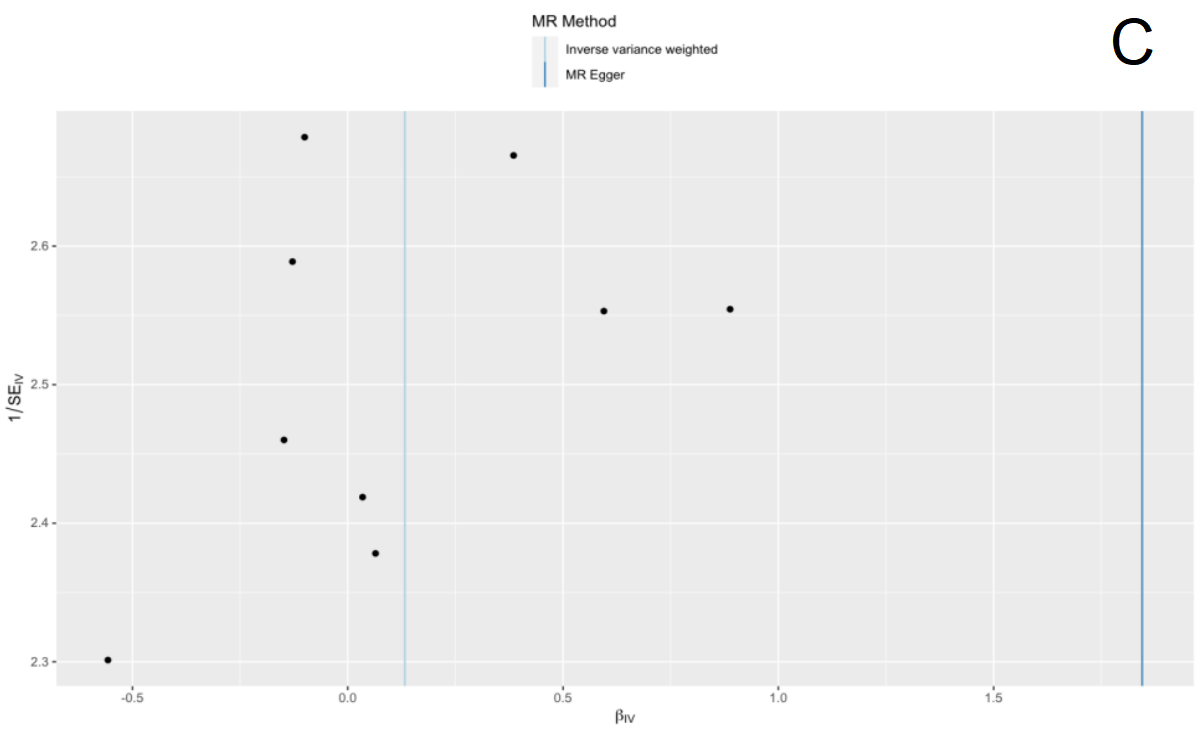

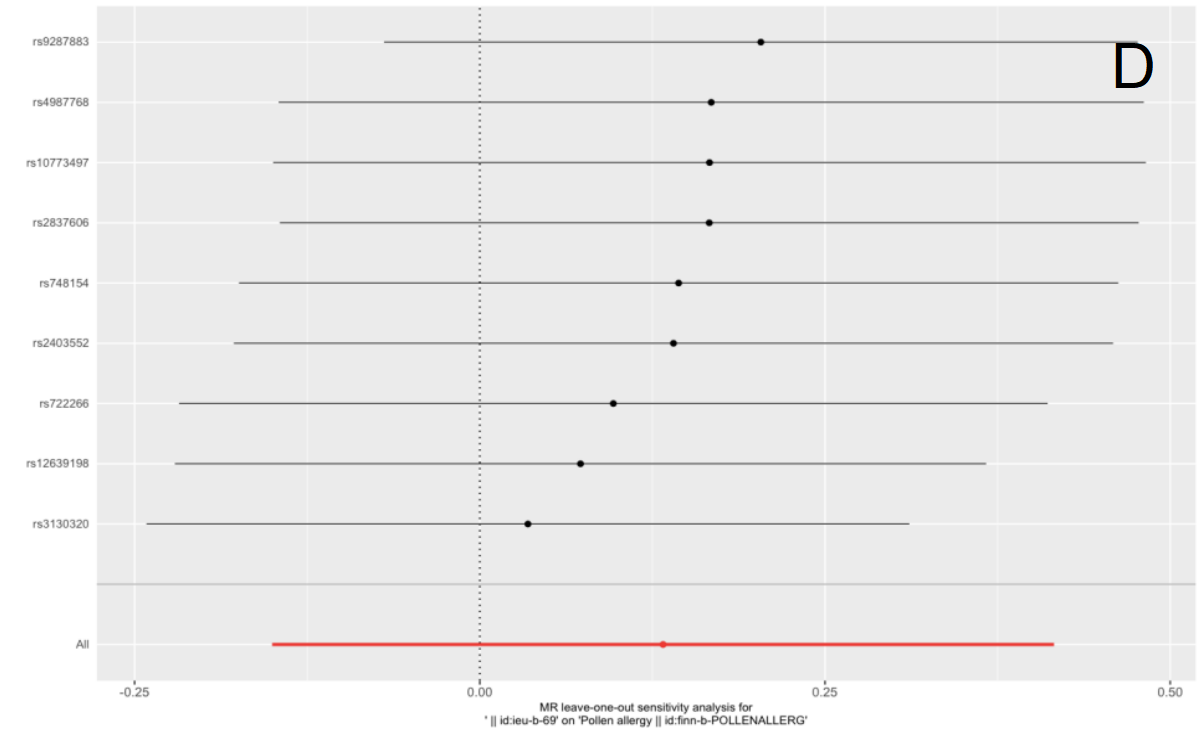


**Supplemental Figure 8 Scatter plot, forest plot, funnel plot and leave-one-out test for adult sepsis affecting allergy**

Figure 8A: Scatter plot; Figure 8B: Forest plot; Figure 8C: Funnel plot; Figure 8D: Leave-one-out test


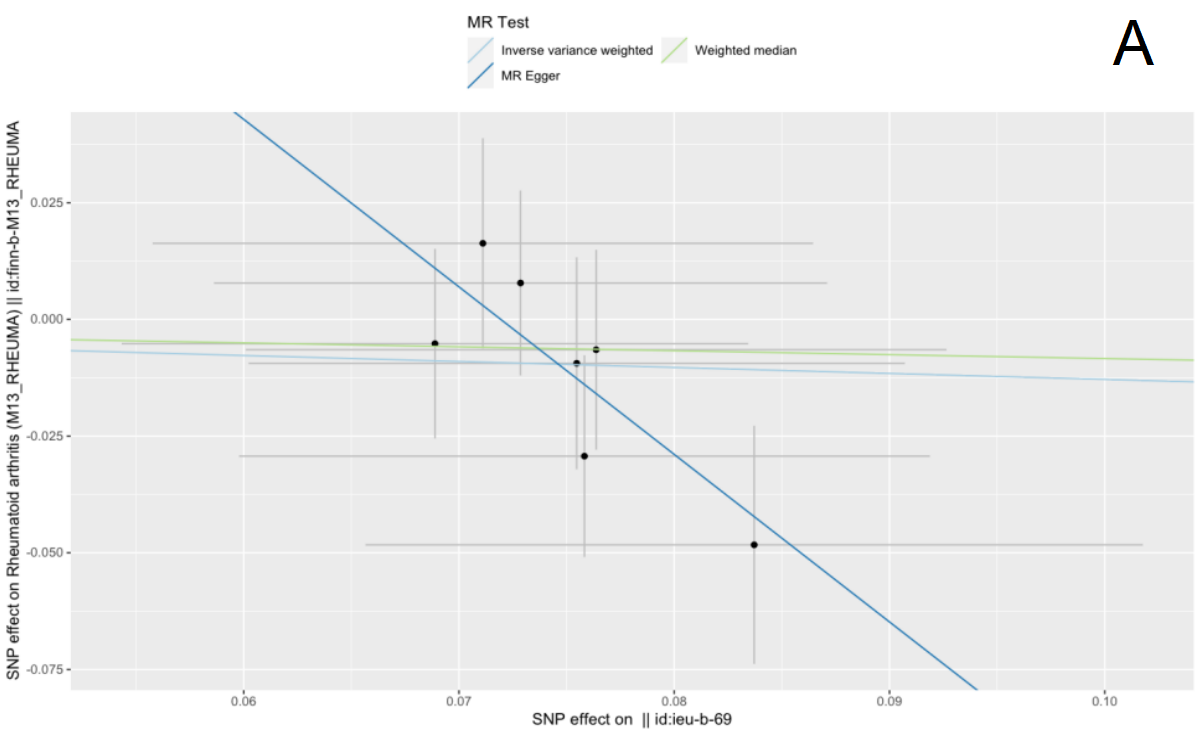

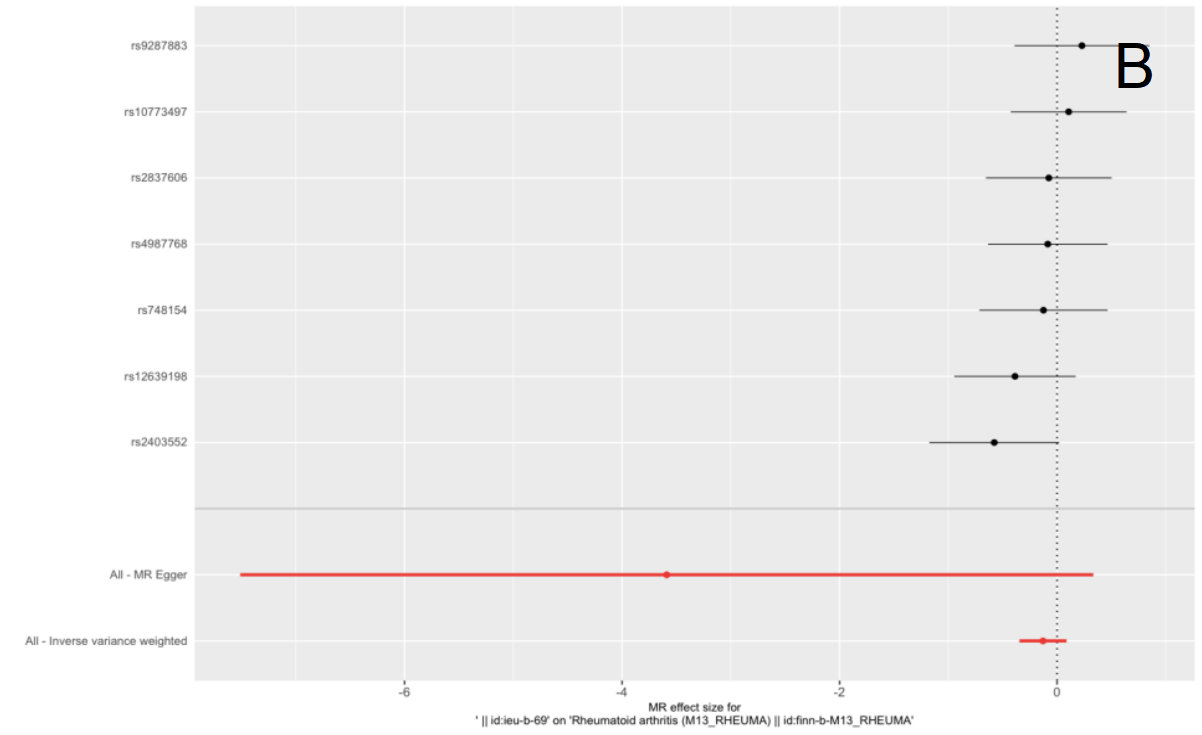


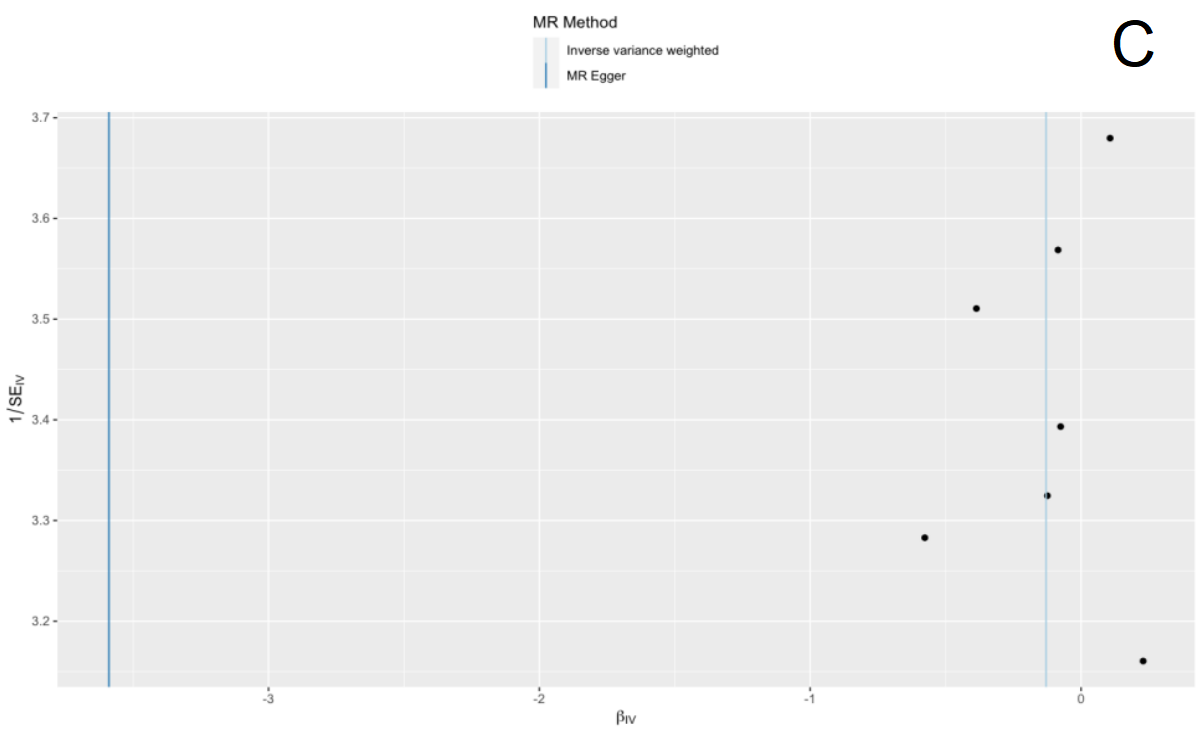

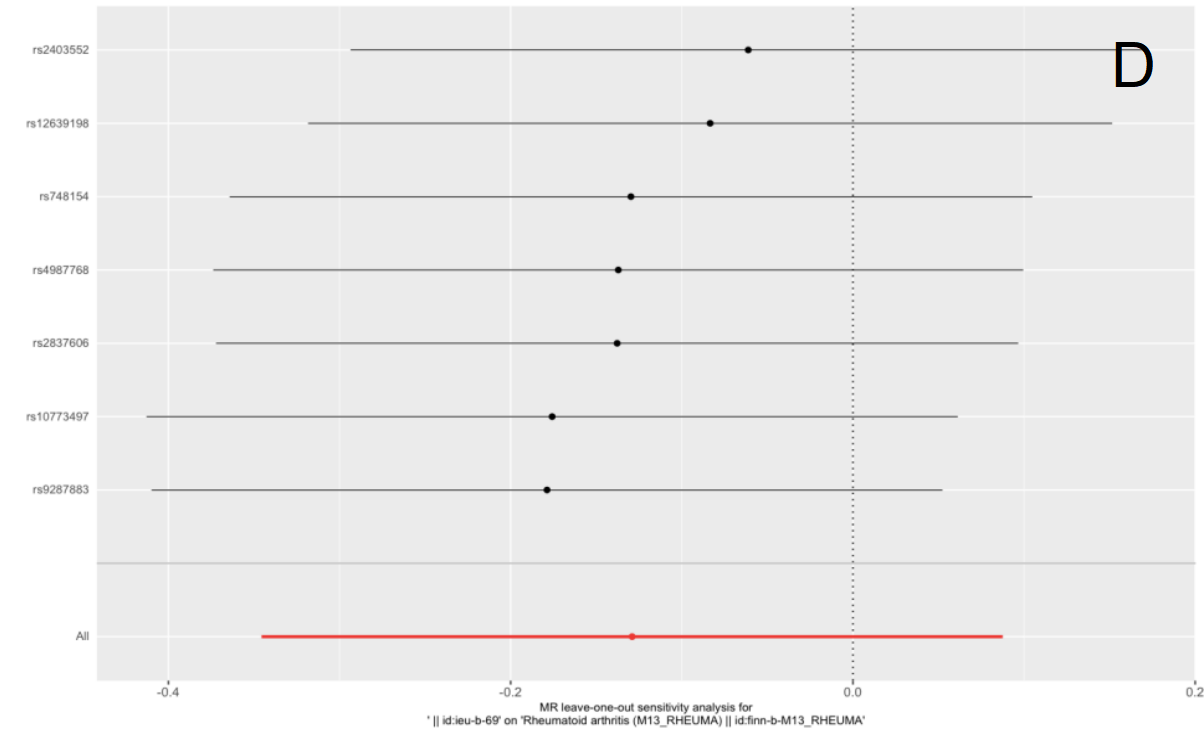


**Supplemental Figure 9 Scatter plot, forest plot, funnel plot and leave-one-out test for adult sepsis affecting rheumatoid arthritis**

Figure 9A: Scatter plot; Figure 9B: Forest plot; Figure 9C: Funnel plot; Figure 9D: Leave-one-out test


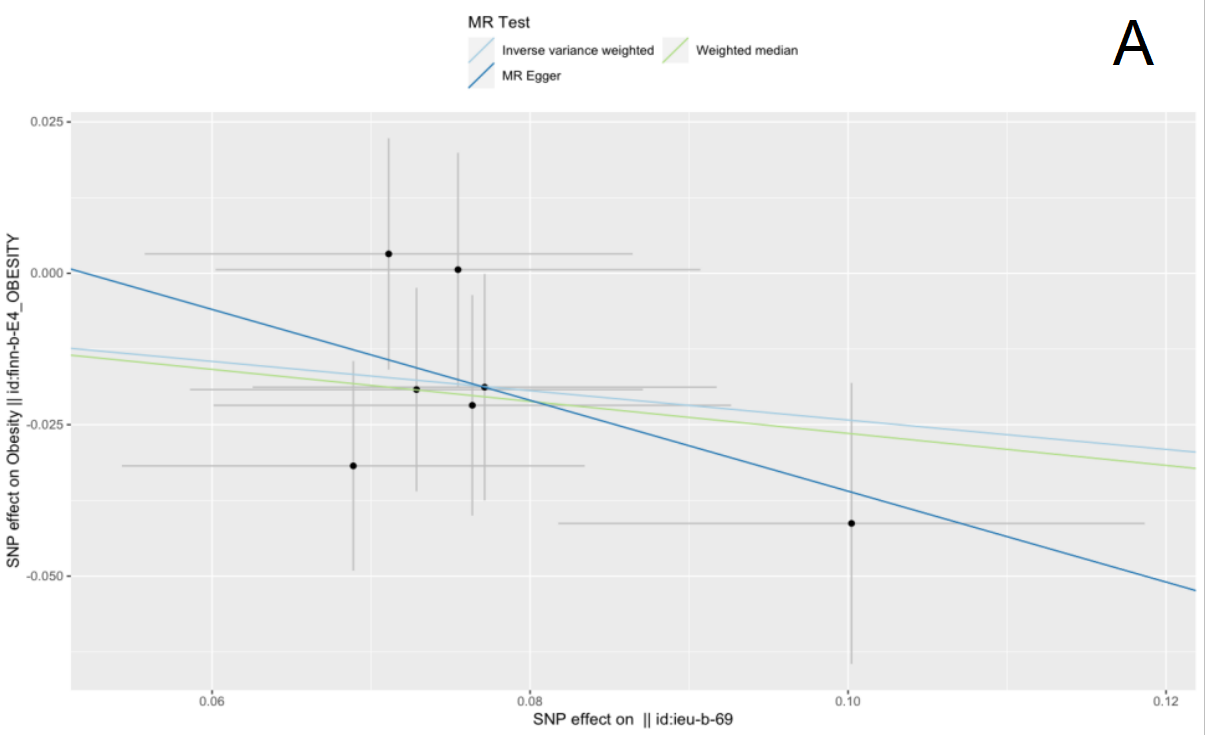

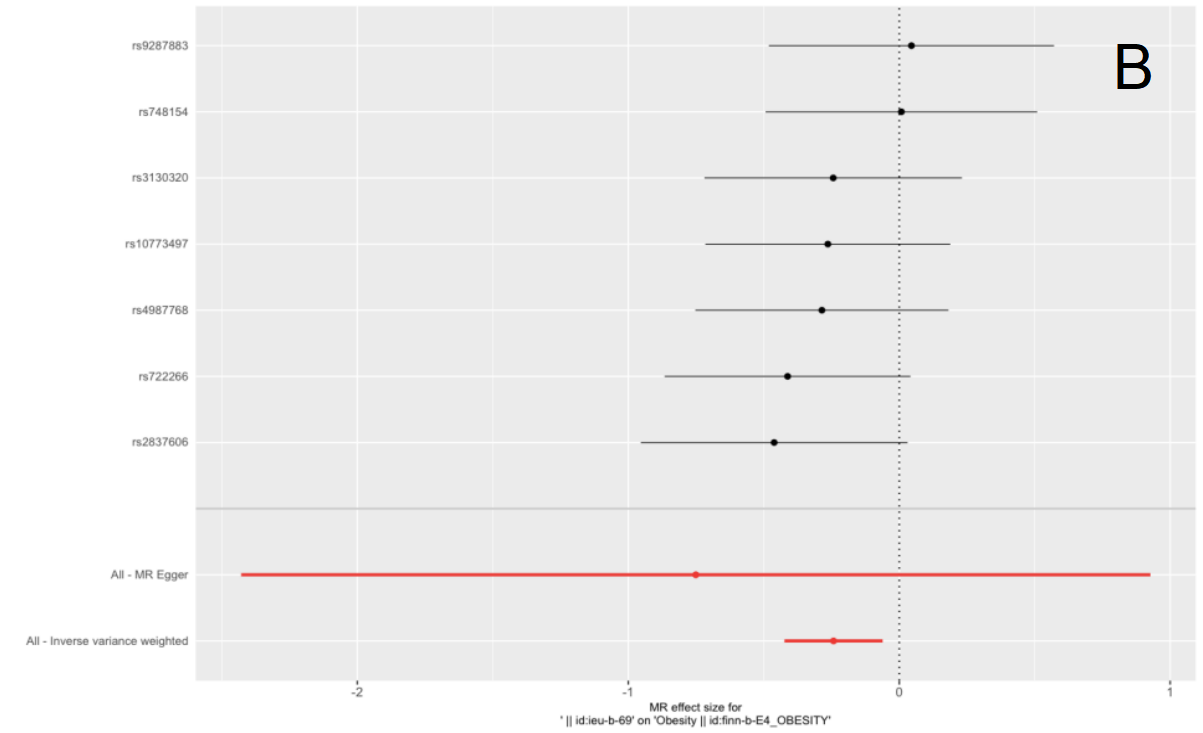


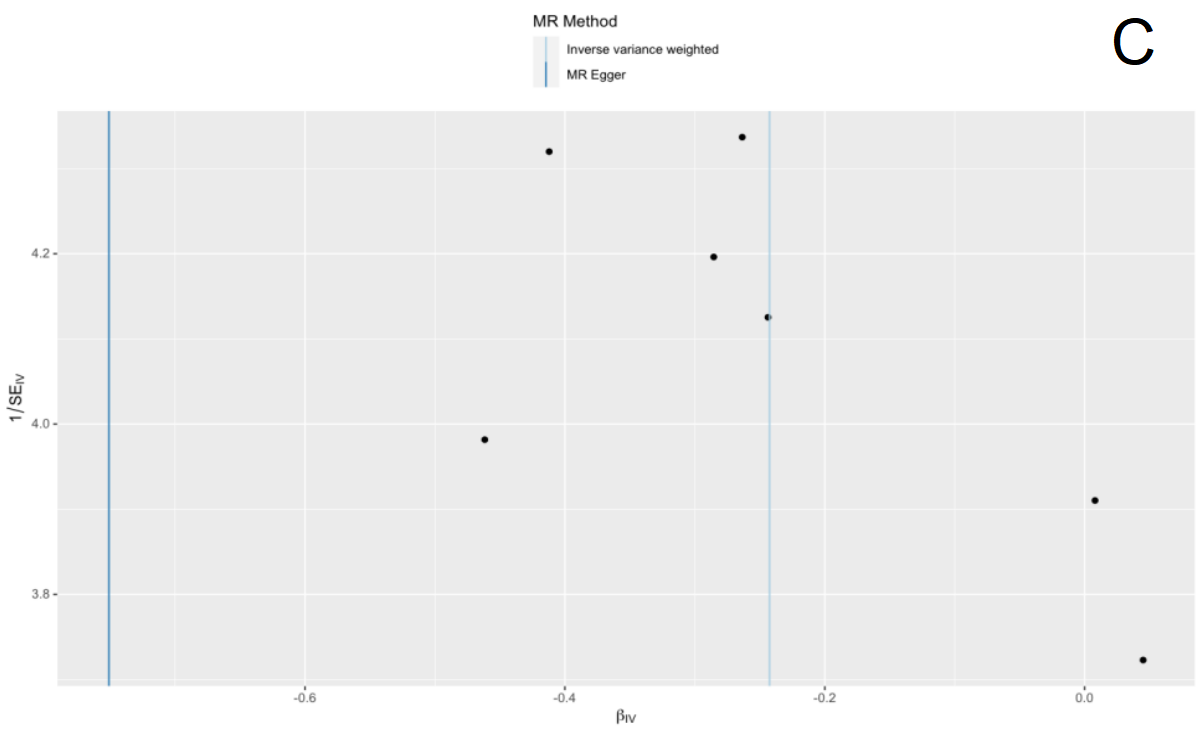

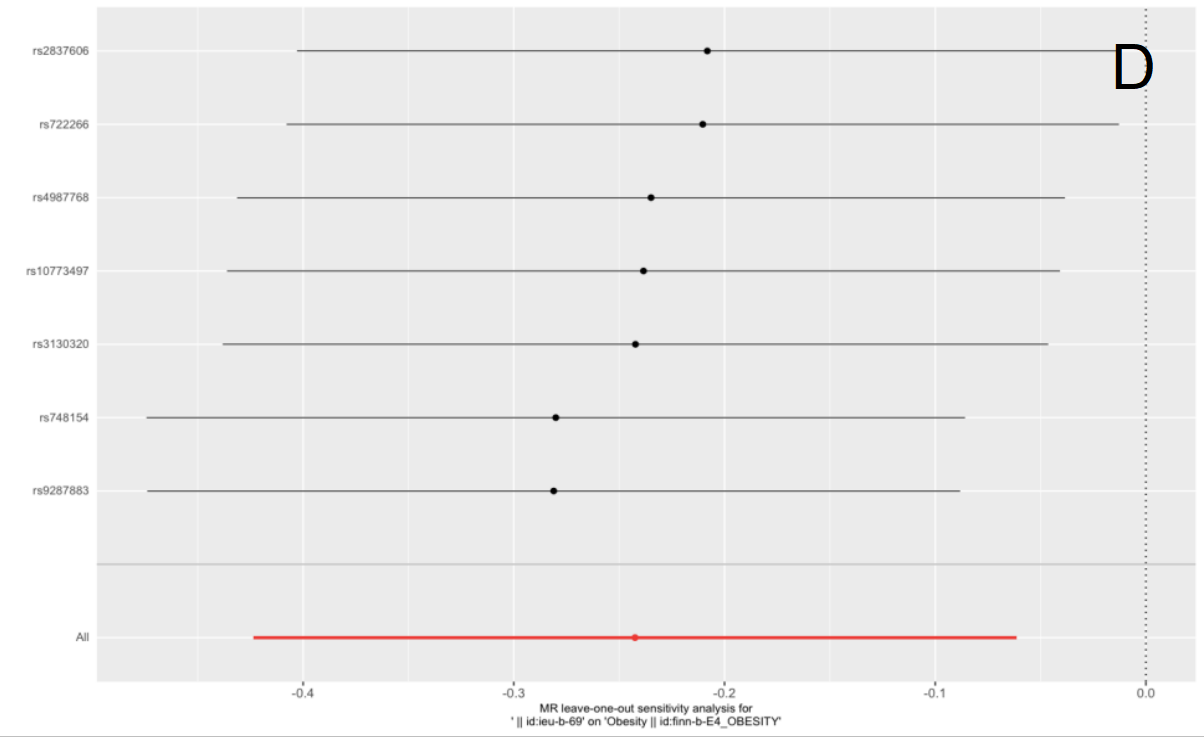


**Supplemental Figure 10 Scatter plot, forest plot, funnel plot and leave-one-out test for adult sepsis affecting obesity**

Figure 10A: Scatter plot; Figure 10B: Forest plot; Figure 10C: Funnel plot; Figure 10D: Leave-one-out test


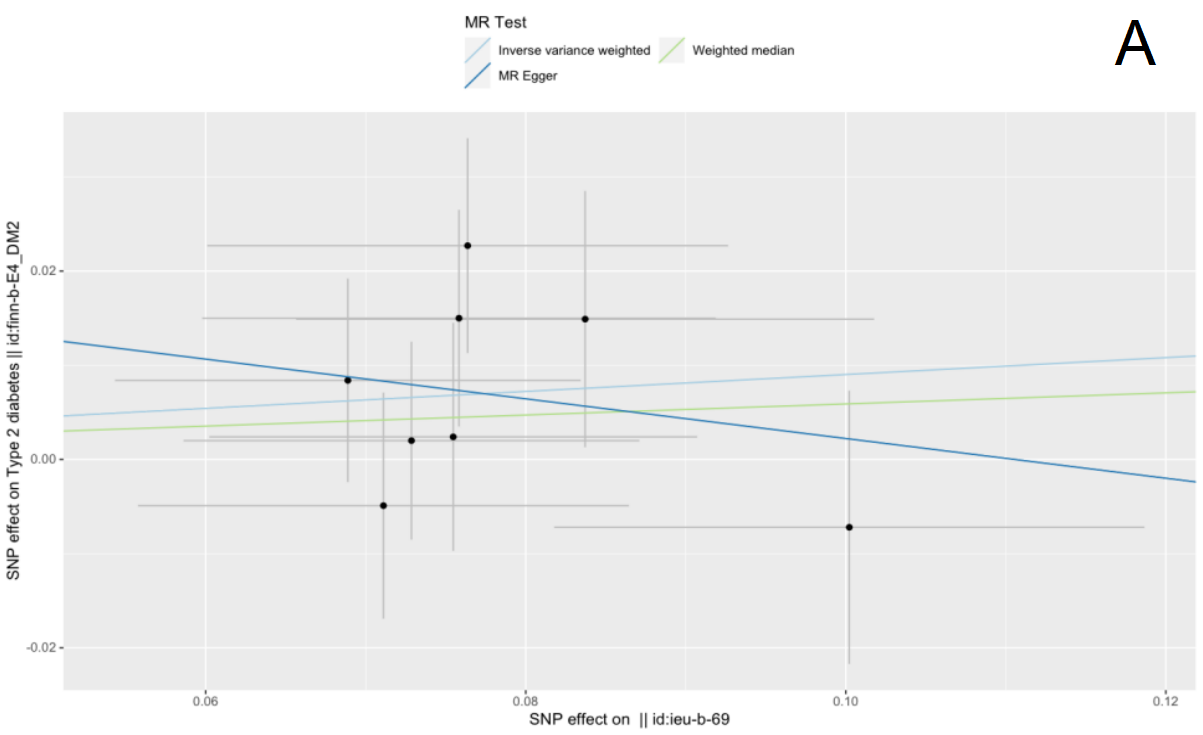

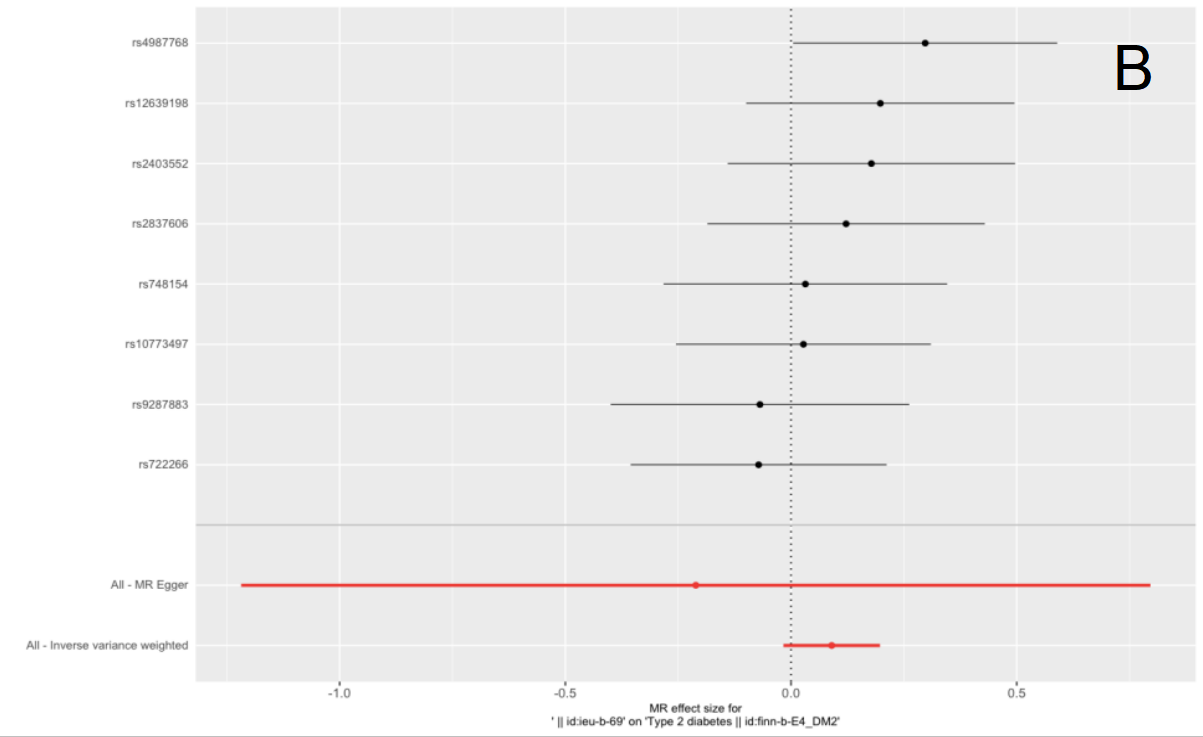


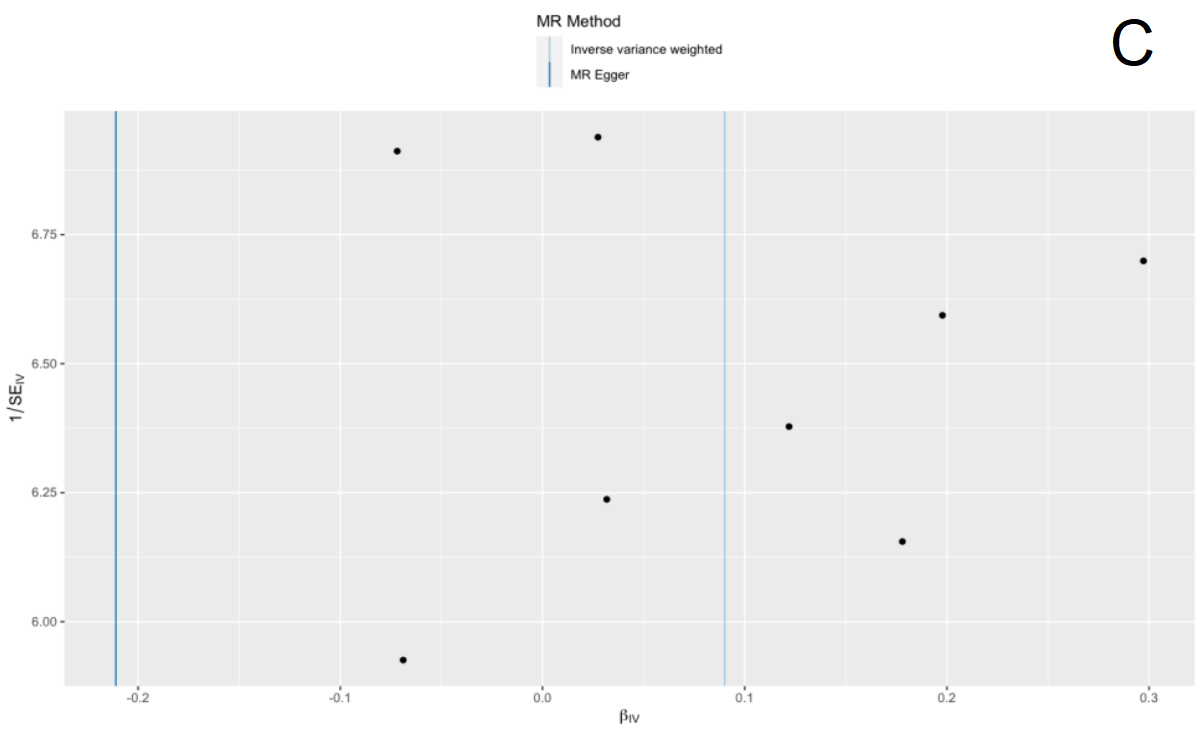

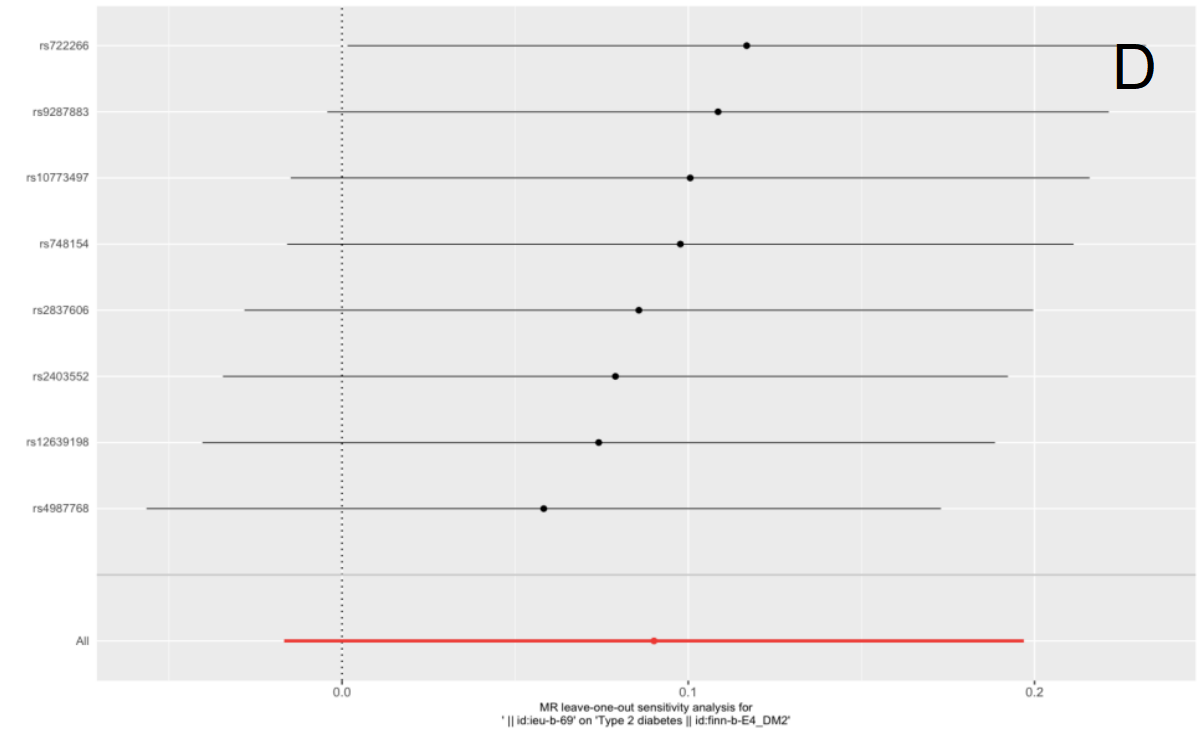


**Supplemental Figure 11 Scatter plot, forest plot, funnel plot and leave-one-out test for adult sepsis affecting type 2 diabetes**

Figure 11A: Scatter plot; Figure 11B: Forest plot; Figure 11C: Funnel plot; Figure 11D: Leave-one-out test


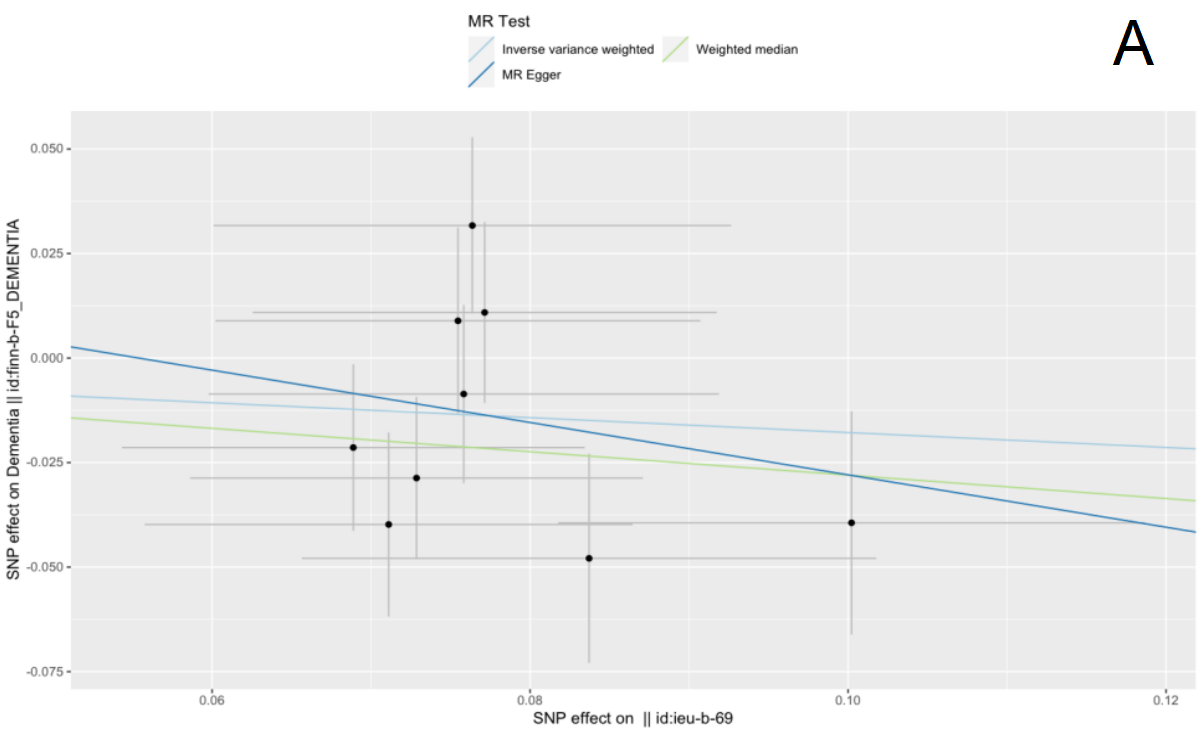

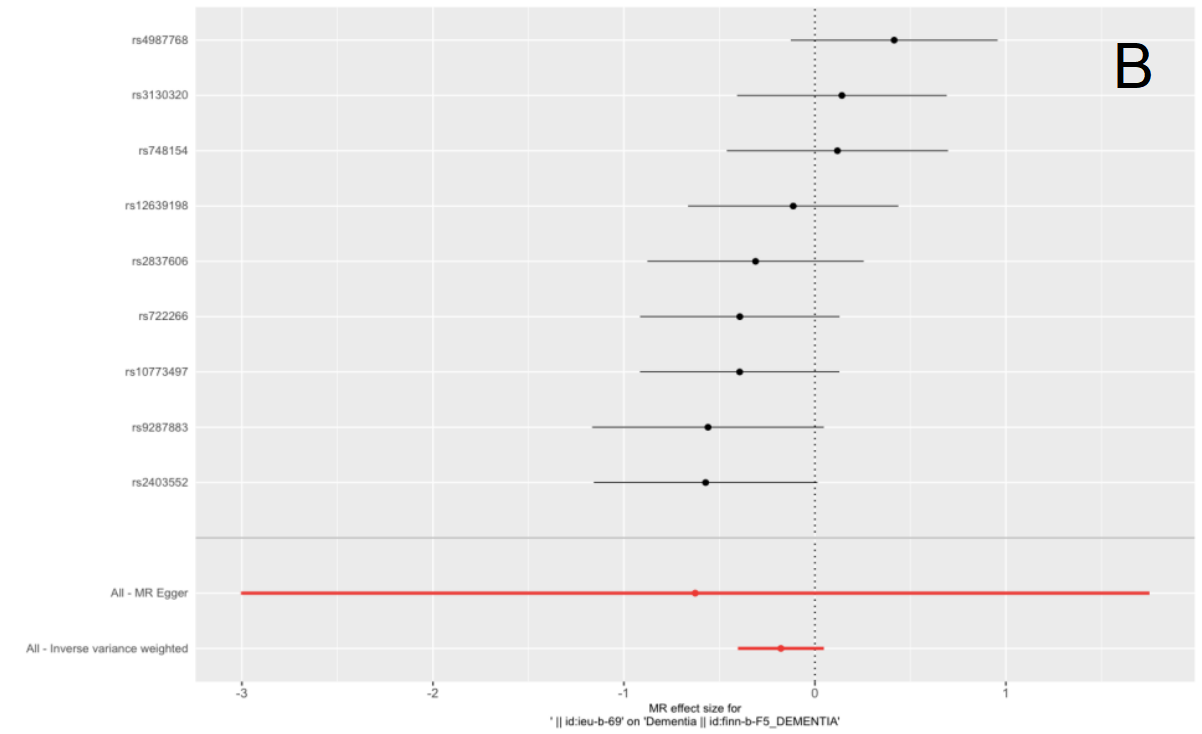


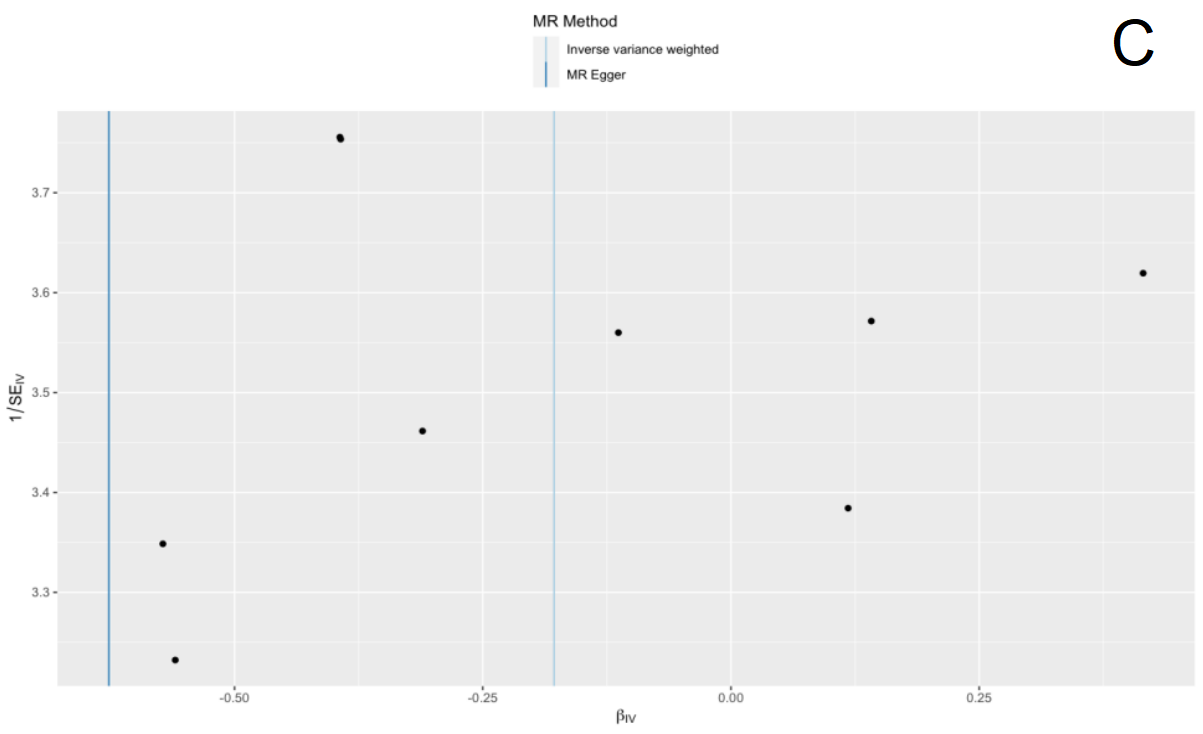

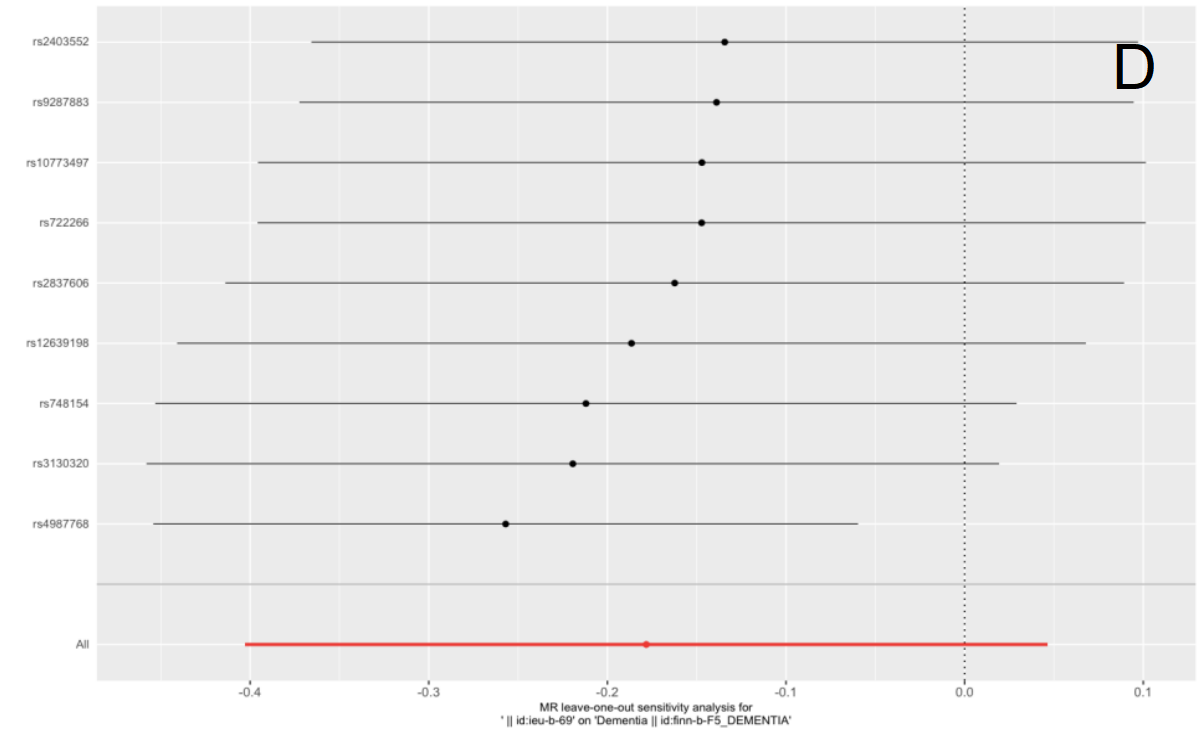


**Supplemental Figure 12 Scatter plot, forest plot, funnel plot and leave-one-out test for adult sepsis affecting dementia**

Figure 12A: Scatter plot; Figure 12B: Forest plot; Figure 12C: Funnel plot; Figure 12D: Leave-one-out test
